# Exploring Causal Associations Between Serum Inflammatory Markers and Female Reproductive Disorders: A Mendelian Randomisation Study

**DOI:** 10.3390/biom14121544

**Published:** 2024-12-02

**Authors:** Simon Alesi, Helena Teede, Lisa Moran, Joanne Enticott, Kushan De Silva, Aya Mousa

**Affiliations:** 1 Monash Centre for Health Research and Implementation (MCHRI), Monash University, Clayton, VIC 3800, Australialisa.moran@monash.edu (L.M.);; 2 Department of Radiation Sciences, Faculty of Medicine, Umeå University, SE-901 87 Umeå, Sweden; kushan.ranakombu@umu.se

**Keywords:** mendelian randomisation, inflammation, endometriosis, polycystic ovary syndrome (PCOS), interleukins, monocyte chemoattractant protein-1/C-C motif ligand-2 (MCP-1/CCL2)

## Abstract

Although inflammation may disrupt immunoendocrine crosstalk essential for female reproductive function, causal links to disorders like polycystic ovary syndrome (PCOS) and endometriosis remain unestablished. This study aimed to utilise Mendelian randomisation (MR) methods to explore causal associations between serum inflammatory markers and common reproductive disorders, aiming to identify novel mechanisms and potential avenues for treatment. Total causal effects of serum inflammatory markers (interleukins, monocyte chemoattractant protein-1, etc.) on female reproductive disorders in large sample cohorts of Finnish ancestry were assessed using univariable two-sample MR methods, including the inverse variance weighted (IVW) method as the primary analysis, with relevant quality assessments (e.g., leave-one out, heterogeneity, and horizontal pleiotropy testing). The main outcome measures were PCOS (642 cases and 118,228 controls) and endometriosis (8288 cases and 68,969 controls) from the FINNGEN cohort. Monocyte chemoattractant protein-1/C-C motif chemokine ligand demonstrated a positive causal association with polycystic ovary syndrome (odds ratio [95% CI]: 1.48 [1.10, 2.00], *p* = 0.0097), while higher interleukin-9 levels were positively associated with endometriosis (1.15 [1.02, 1.30], *p* = 0.0277), both via the IVW method. These markers should be investigated as key candidates for future research into the mechanistic pathways underpinning these conditions.

## 1. Introduction

Polycystic ovary syndrome (PCOS) and endometriosis are the most prevalent female reproductive disorders globally and are among the leading causes of infertility in women [1]. PCOS affects 10–13% of women of reproductive age and is diagnosed by International Guideline criteria using two of irregular menstrual cycles, clinical or biochemical hyperandrogenism, and/or polycystic ovary morphology or anti-Mullerian hormone levels [2]. PCOS has also been shown to increase the risk of metabolic (obesity, type 2 diabetes, cardiovascular diseases), psychological (anxiety and depression), dermatological (hirsutism and acne), and reproductive (infertility, pregnancy complications, and endometrial cancer) conditions [2]. Despite its lifelong impact, 70% of women with PCOS remain undiagnosed, owing in large part to its complex and poorly understood aetiology [2]. Similarly, endometriosis is another common and complex disorder, affecting 10–15% of reproductive-aged women and girls globally [3] and up to 50% of women with infertility [4]. Diagnosed by a combination of symptoms assessment, pelvic exam, imaging studies, and/or laparoscopic surgery, this chronic condition is characterised by endometrial-like tissue growth outside the uterine cavity, primarily affecting pelvic structures including the ovary and fallopian tubes [3]. Together, PCOS and endometriosis form a substantial portion of the global female reproductive burden, highlighting the importance of understanding the causal pathways of these disorders to optimise treatment approaches.

Although these conditions are distinct and multifactorial, a common feature in both PCOS and endometriosis is the presence of chronic low-grade inflammation [5,6]. In PCOS, the tetradic interplay of insulin resistance, hyperandrogenism, sympathetic dysfunction, and chronic inflammation is thought to promote a pathological milieu that fosters reproductive and cardiometabolic disturbances [7,8]. In endometriosis, ectopic tissue growth induces a chronic inflammatory state, which can lead to fertility problems, fibrous adhesions, and frequent, often debilitating, pelvic pain [9]. Despite evidence that inflammation may disrupt the immunoendocrine crosstalk critical for normal female reproductive function [10], causal links between inflammation and these reproductive disorders have not been established. To date, the majority of human studies examining inflammation in reproductive disorders have relied on observational data, which are limited by inadequate matching, uncontrolled confounding, and other biases, precluding causation [11].

Mendelian randomisation (MR) is a powerful statistical method that leverages genetic variant data to assess causal pathways of disease. This method provides a unique advantage in establishing causal inferences, analogous to the benefits offered by randomised controlled trials (RCTs) without the added costs or time investments [12]. Using genetic variants as instrumental variables and applying Mendel’s laws of segregation and random assortment [13], MR offers a practical means of plausibly deducing causality between exposures (risk factors) and outcomes (diseases or traits) [12]. As such, MR can leverage observational data while circumventing common biases introduced by confounding or other measurement errors in traditional observational studies. To date, however, MR studies examining inflammatory markers in female reproductive disorders are sparse and inconclusive [refs], highlighting the need for rigorous MR studies to clarify the causal mechanisms underpinning these disorders.

In this study, we hypothesised that heightened levels of circulating pro-inflammatory markers are causally associated with female reproductive conditions. To test this hypothesis, we aimed to investigate whether there are causal associations between inflammatory marker exposures and female reproductive disorders (PCOS and endometriosis) using robust MR analysis techniques.

## 2. Materials and Methods

This study is reported according to the Strengthening the Reporting of Observational Studies in Epidemiology using Mendelian randomisation (STROBE-MR) guidelines [14,15]. All analyses were conducted on de-identified, summarised, and publicly available data; hence, formal ethics approval was not required.

### 2.1. Data Sources for Inflammatory Marker Exposures

Serum inflammatory markers were selected as exposures based on biological plausibility and the literature on relevance to female reproductive disorders. These included C-reactive protein (CRP), monocyte chemoattractant protein-1/C-C motif ligand-2 (MCP-1/CCL2), various interleukins (ILs), tumour necrosis factor-alpha (TNF-α), and interferon-γ (IFN-γ). A comprehensive search of Genome-Wide-Association-Study (GWAS) data from the Integrative Epidemiology Unit (IEU) Project database (https://gwas.mrcieu.ac.uk/) was conducted in order to determine the most appropriate GWAS datasets for this MR analysis (i.e., data with large sample sizes and various associated single nucleotide polymorphisms [SNPs]). Details of the retrieved studies (including citations and GWAS IDs) are displayed in Table 1. Briefly, the selected GWAS datasets for all markers were from meta-analyses. For CRP, we used age- and sex-adjusted data from a large-scale meta-analysis (16,540 individuals from 10 independent studies), the specific details of which are described elsewhere [16]. The remaining markers were from meta-analyses from three independent cohorts: the Cardiovascular Risk in Young Finns Study cohort and two National FINRISK Study cohorts: FINRISK1997 and FINRISK2002, all collectively comprising 8,293 Finnish participants. These data were adjusted for age, sex, and body mass index, as described in the original study [17].

### 2.2. Data Source for Female Inflammatory Condition Outcomes

Outcomes of interest were those related to the most common female reproductive conditions: PCOS and endometriosis. To match the origins of the outcome and exposure databases and maximise cohort sizes for each outcome, the FinnGen outcome database was selected. This includes genomic and clinical data from a network of national Finnish biobanks (https://finngen.gitbook.io/documentation/ (accessed on 15 February 2024)). Specific GWAS IDs used and their corresponding phenocodes are outlined in Table 1. Using this database, the outcome of PCOS was available for 118,870 individuals (n = 642 cases and 118,228 controls), resulting in 16,379,676 genotyped SNPs. Endometriosis outcome data were available for 77,257 individuals (n = 8288 cases and 68,969 controls), totalling 16,377,306 genotyped SNPs. Outcomes related to PCOS and endometriosis were identified through hospital discharge records and cause of death registries using female-specific clinical endpoints.

### 2.3. Selection of Genetic Variants as Instrumental Variables

Mendel’s laws of independent assortment and segregation of alleles [18] suggest that, to ensure valid MR results, the SNPs selected as instrumental variables must fulfil three key assumptions [19]. These assumptions specify that the instrumental variable must: (i) be strongly associated with the exposure (i.e., relevance); (ii) not be associated with the outcome due to confounding (i.e., independence); (iii) only affect the outcome through the exposure and be on the causal pathway (i.e., exclusion restriction) [19]. To assess weak instrument bias, we used the F-statistic, where F > 10 indicates no weak instrument bias. The F-statistic was calculated following the methodology outlined elsewhere [20]. To satisfy the first criteria of relevance, we selected SNPs at a significance threshold *p* < 5 × 10^−6^, a threshold considered to have sufficient specificity and equivalent sensitivity to stricter thresholds, particularly when using sufficiently large sample sizes (n > 20,000) [21,22,23]. We also assessed the non-random association between alleles of genetic loci that lie in close proximity with one another by testing the linkage disequilibrium (LD) (13), the absence of which is a pre-requisite for MR analysis. Here, we performed clumping to prune statistically plausible independent variables, selecting SNPs with LD-R2 < 0.01 and clumping distance > 10,000 kB. The remaining criteria of independence and exclusion restriction were assessed by the quality assessments described below (e.g., leave-one-out, heterogeneity, and horizontal pleiotropy).

### 2.4. Two-Sample MR Analyses

Statistical analyses were conducted in RStudio v4.2.0 using the packages TwoSampleMR, MRPracticals, and MRInstruments. Five distinct two-sample MR (2-SMR) methods were applied. The first of these is the inverse variance weighted (IVW) method, considered the primary analysis. As the central method used for analysing summarised GWAS data, this approach utilises multiple, uncorrelated genetic variants and a multiplicative random effects model and is therefore considered the most robust approach in the presence of valid instrumental variables [24].

The four additional methods include MR-Egger (MRE), weighted median (WMe), weighted mode (WMo), and simple mode (SMo) [24]. The MRE method accounts for horizontal pleiotropy and provides an asymptotically consistent measure of causal effect, achieved by pooling SNP-specific Wald ratios. The WMe method also yields consistent causal effect estimates but requires that >50% of the instrumental variables are valid and satisfy the ‘exclusion restriction’ criterion. This criterion requires that any effect of the proposed instrument on the outcome is exclusively through the effect on the exposure, though this cannot be definitively verified [24]. The WMo method clusters the various SNPs based on similarities between ratio estimates and assumes that the most common causal effect is the true causal effect [25]. Finally, in the SMo method, SNPs are clustered based on similarities in causal effect estimates, determining the causal effect from the group with the highest SNP count [25].

### 2.5. Method Comparison Plots

Scatter plots and trend-lines related to the five two-sample MR methods described above (IVW, MRE, WMe, WMo, and SMo) were generated for each serum inflammatory exposure-reproductive condition outcome analysis. The gradient and direction of the trend-line represent the magnitude and direction of the causal effect, respectively.

### 2.6. Single SNP Analyses

Forest plots were used to visualise the causal effects of each SNP and the pooled estimates under the IVW and MRE methods. A Bonferroni correction was applied to account for multiple testing, given the number of individual SNPs included in the analysis.

### 2.7. Leave-One-Out Sensitivity Analyses

To examine whether the effect estimate varied by iterative omission of any single SNP, leave-one-out sensitivity analyses were conducted.

### 2.8. Heterogeneity Analyses

The heterogeneity of causal associations was assessed to verify the core assumption of MR homogeneity (the effect of the exposure on the outcome is the same for all individuals in the cohort). This was tested using Cochran’s Q statistic and associated *p*-values with funnel plots generated to visually assess heterogeneity.

### 2.9. Analysis of Horizontal Pleiotropy and Outliers

Across all associations between inflammatory exposures and reproductive conditions, we analysed the MRE regression intercept to estimate the magnitude of horizontal pleiotropy (a single locus affecting two or more seemingly unrelated phenotypic traits) and the presence of outliers.

## 3. Results

### 3.1. Two-Sample MR (2-SMR) Results

There is no evidence of weak instrument bias (F > 10; Supplementary Data File S3). Selected results of 2-SMR analyses are presented in Table 2, with full results presented in Supplementary Table S1. In the primary analysis using the IVW method, there was a significant positive causal association between MCP-1/CCL2 and PCOS. Here, a standard deviation (SD) unit increase in MCP-1/CCL2 corresponded with increased odds of having PCOS, with an odds ratio (OR) of 1.48 (95% CI: 1.10, 2.00; *p* = 0.0097; Table 2). Also using the IVW method, an SD unit increase in IL-9 was significantly associated with increased odds of endometriosis (OR [95% CI]: 1.15 [1.02, 1.30], *p* = 0.0277; Table 2). Using the WMe method, there was also a positive causal association for IL-2 with endometriosis (OR [95% CI]: 1.18 [1.01, 1.37], *p* = 0.0352; Table 2).

### 3.2. Method Comparison Plots

**Figure 1:**
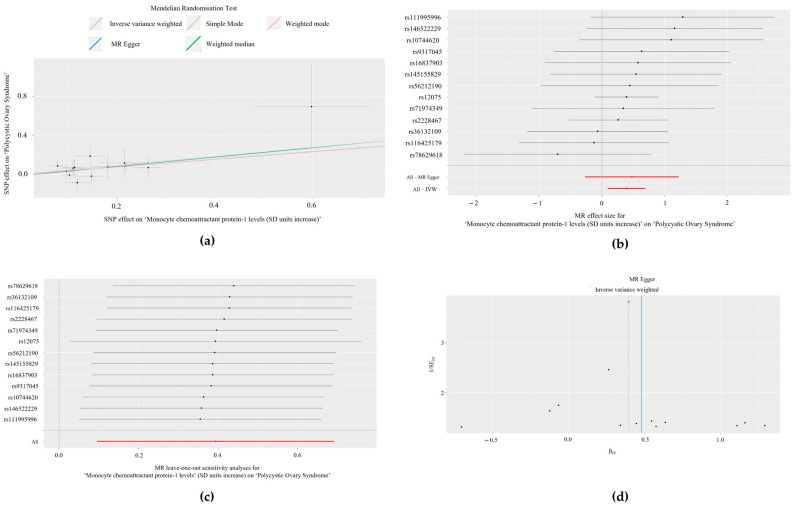
The relationship between ‘monocyte chemoattractant protein-1 (MCP-1/CCL2) (standard deviation units increase)’ and the outcome of ‘polycystic ovary syndrome’, visualised by: (**a**) Scatter plot denoting the distribution of individual causal estimates, with lines indicating the trendlines of the causal estimate using each method; (**b**) Forest plot of the effects of individual single nucleotide polymorphisms (SNPs) and pooled estimates visualised via mendelian randomisation-egger (MRE) and inverse variance weighted (IVW) methods; (**c**) Leave-one-out analysis plot, where the dark points indicate the effect measure via IVW-mendelian randomisation (IVW-MR) analysis excluding the specific SNP. The red line indicates the pooled analysis encompassing all SNPs via IVW-MR; and (**d**) Funnel plot to assess heterogeneity.

**Figure 2:**
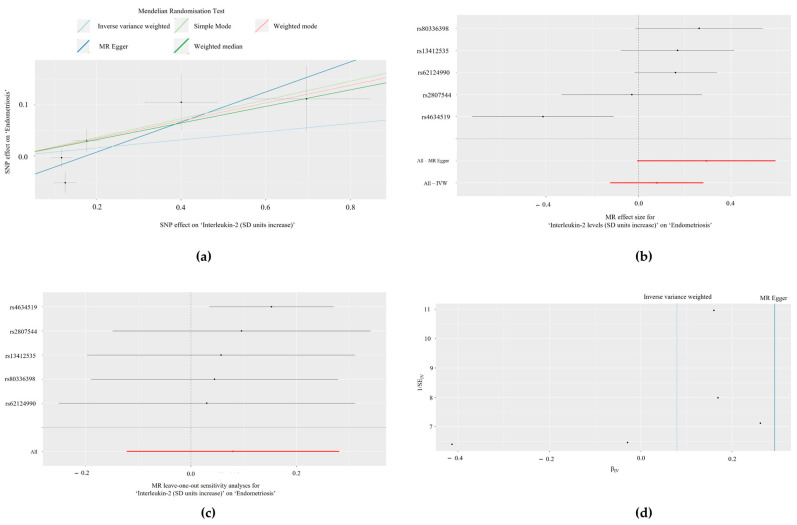
The relationship between ‘interleukin-2 (IL-2) (standard deviation units increase)’ and the outcome of ‘endometriosis’, visualised by: (**a**) Scatter plot denoting the distribution of individual causal estimates, with lines indicating the trendlines of the causal estimate using each method; (**b**) Forest plot of the effects of individual single nucleotide polymorphisms (SNPs) and pooled estimates visualised via mendelian randomisation-egger (MRE) and inverse variance weighted (IVW) methods; (**c**) Leave-one-out analysis plot, where the dark points indicate the effect measure via IVW-mendelian randomisation (IVW-MR) analysis excluding the specific SNP. The red line indicates the pooled analysis encompassing all SNPs via IVW-MR; and (**d**) Funnel plot to assess heterogeneity.

**Figure 3:**
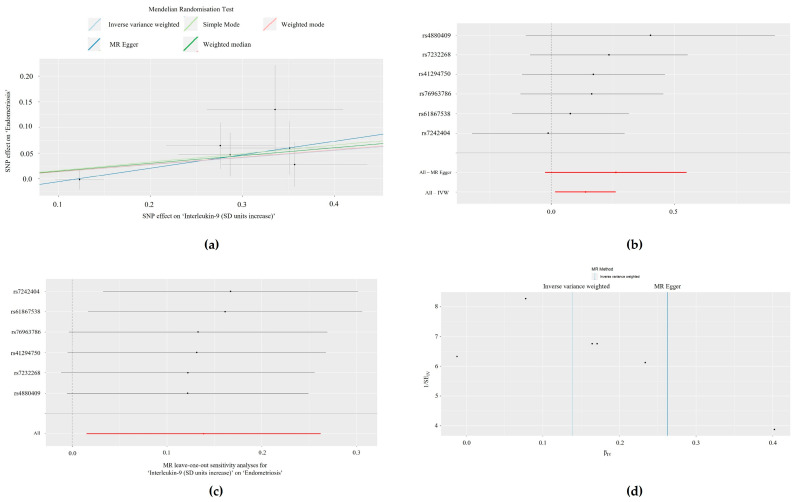
The relationship between ‘interleukin-9 (IL-9) (standard deviation units increase)’ and the outcome of ‘endometriosis’, visualised by: (**a**) Scatter plot denoting the distribution of individual causal estimates, with lines indicating the trendlines of the causal estimate using each method; (**b**) Forest plot of the effects of individual single nucleotide polymorphisms (SNPs) and pooled estimates visualised via mendelian randomisation-egger (MRE) and inverse variance weighted (IVW) methods; (**c**) Leave-one-out analysis plot, where the dark points indicate the effect measure via IVW-mendelian randomisation (IVW-MR) analysis excluding the specific SNP. The red line indicates the pooled analysis encompassing all SNPs via IVW-MR; and (**d**) Funnel plot to assess heterogeneity.

Method comparison plots are presented consisting of scatter plots and trendlines for the associations between MCP-1/CCL2 with PCOS (Figure 1a) and between ILs-2 and -9 with endometriosis (Figure 2a and Figure 3a, respectively).

### 3.3. Single SNP Analyses

Forest plots displaying single SNP analyses and pooled causal estimates via the IVW and MRE methods are presented in Figure 1b for the relationship between MCP-1/CCL2 with PCOS (Figure 1b) and ILs-2 and -9 with endometriosis (Figure 2b and Figure 3b, respectively). In the Bonferroni-corrected analysis, no single SNPs were individually associated with PCOS (MCP-1/CCL2: *p* > 3.85 × 10^−3^, 0.05/13). However, one SNP was individually associated with endometriosis in the IL-2 (rs4634519, *p* = 0.0082; *p* < 0.01, 0.05/5), but not IL-9 analysis (*p* > 8.33 × 10^−3^, 0.05/6). The results of all single SNP analyses are presented in Supplementary Data File S1.

### 3.4. Leave-One-Out Analyses

Results from leave-one-out analyses are presented for the associations between MCP-1/CCL2 with PCOS (Figure 1c) and ILs-2 and -9 with endometriosis (Figure 2c and Figure 3c, respectively). In Bonferroni-corrected leave-one-out analyses, omitting single SNPs did not achieve statistical significance for exposures associated with PCOS (MCP-1/CCL2: *p* > 3.85 × 10^−3^, 0.05/13) or endometriosis (IL-2: *p* > 0.01, 0.05/5; IL-9: *p* > 8.33 × 10^−3^, 0.05/6). The results of all leave-one-out analyses are presented in Supplementary Data File S2.

### 3.5. Heterogeneity Analyses

Heterogeneity analyses (Cochran’s Q and *p*-values) for significant exposures (MCP-1/CCL2, IL-2, and IL-9) are summarised in Table 3. There was no statistically significant heterogeneity in any 2-SMR analyses (*p* > 0.05), except for IL-2 and endometriosis, which displayed statistically significant heterogeneity via the IVW method. These results are presented in funnel plots for the associations between MCP-1/CCL2 and PCOS (Figure 1d) and between ILs-2 and -9 and endometriosis (Figure 2d and Figure 3d).

### 3.6. Horizontal Pleiotropy and Outliers

Horizontal pleiotropy (MRE intercepts and directionality *p*-values) for significant exposures (MCP-1/CCL2, IL-2, and IL-9) are summarised in Table 4. There was no significant horizontal pleiotropy for any of these markers (all *p* > 0.05).

## 4. Discussion

### 4.1. Main Findings

To our knowledge, this is the first MR study to demonstrate positive causal associations between CXCL2/MCP1 and PCOS using the primary IVW method. We also found that circulating IL-9 was positively causally associated with endometriosis in IVW analysis. Although IL-2 demonstrated an association with endometriosis using WMe analysis, this did not fulfil the MR quality assessments due to significance in single SNP analysis and heterogeneity. Nevertheless, our novel findings have highlighted key inflammatory markers that should be investigated further in the context of PCOS and endometriosis.

### 4.2. Interpretation

Our finding of a causal association between MCP-1/CCL2 and PCOS generated an odds ratio of 1.48 using the IVW method. MCP-1/CCL2 is a crucial chemotactic factor that triggers the recruitment, migration, and infiltration of monocytes/macrophages, basophils, and white blood cells in a variety of different tissue types [26,27]. As a potent initiator of inflammation, MCP-1/CCL2 has been implicated in the development of numerous chronic disorders, including rheumatoid arthritis, cardiovascular diseases, and PCOS [28,29]. While several observational studies have reported an upregulation of MCP-1/CCL2 in women with PCOS compared with controls [30,31,32,33,34], others have reported no significant difference between groups [35,36]. Attempting to address these inconsistencies, Wu et al. [29] conducted a meta-analysis of 11 studies (n = 897; 529 PCOS and 368 controls) and found that MCP-1/CCL2 levels were significantly elevated in those with PCOS compared to non-PCOS populations. These differences have been shown to be independent of age and body mass index [32,34]. Studies have also linked elevated MCP-1 with clinical symptoms of PCOS, including biochemical and clinical hyperandrogenism (hirsutism) [34] and insulin resistance independently of obesity; however, it is unclear whether elevated MCP-1 in PCOS is independent of insulin resistance [37]. Animal models have indicated tissue-specific effects of MCP-1, whereby effects on whole-body insulin resistance are seen with MCP-1 overexpression in adipose tissue [38], but not in skeletal muscle [39]. The origins and molecular processes that lead to elevated MCP-1/CCL2 levels in women with PCOS are not well understood. Polymorphisms in the MCP-1 promoter gene are thought to be associated with PCOS risk through altering transcriptional activity and upregulating MCP-1 expression [33]. These data indicate that MCP-1 not only participates in the inflammatory processes underpinning PCOS but may also play a role in genetic predisposition to PCOS. Further, MCP-1 and its receptor chemokine receptor 2 (CCR2) are expressed in reproductive organs, including the ovary, with a proposed regulatory role in key ovulatory functions including folliculogenesis and ovulation [40]. Indeed, a recent MR study by our group demonstrated a positive causal association between MCP-1/CCL2 and anovulatory infertility (unpublished). Our reports of causal links between MCP-1/CCL2 and both PCOS and anovulatory infertility are of clinical relevance because PCOS is the most common cause of anovulation and the leading cause of infertility globally [29,41]. The present findings, coupled with the extensively reported mechanisms of action, suggest that MCP-1/CCL2 could plausibly be a causal risk factor for PCOS, with important clinical implications on fertility and cardiometabolic risk. Large-scale clinical studies are now needed to determine whether therapeutic targeting of MCP-1/CCL2 and its downstream pathways may be beneficial in the management of PCOS.

In our primary IVW analyses, IL-9 was positively causally associated with endometriosis, with an odds ratio of 1.15. As a pleiotropic cytokine secreted by T-helper (Th)-2, Th-17, regulatory T (Treg) cells, mast cells, and innate lymphoid cells, IL-9 has been primarily examined in relation to mediating immune responses to parasitic infections and in the pathogenesis of Th2-linked immunological pathologies, such as asthma [42,43]. However, there has been renewed interest in the role of IL-9 in other chronic inflammatory or autoimmune conditions, including lupus nephritis, inflammatory bowel disease, and endometriosis [44,45]. Our finding of a causal relationship between IL-9 and endometriosis builds on and extends prior observational data showing that IL-9 levels were higher in the peritoneal fluid of those with endometriosis compared to those without [46] and were overexpressed in cervicovaginal fluid in the presence of endometriosis [47]. Tarumi et al. [45] also reported that IL-9-producing Th cells (but not IL-9 concentrations) were aberrantly higher in the peritoneal fluid of those with endometriosis compared with controls and that it additively stimulated IL-8 expression in the presence of TNF-a in ovarian endometrioma stromal cells. Since IL-8 contributes to the adhesion, invasion, and proliferation of endometriotic lesions, it was posited that IL-9 indirectly contributes to the development or progression of endometriosis by inducing IL-8 expression. This may occur via phosphorylation of the extracellular signal-regulated kinase (ERK) 1/2 or p38-mitogen-activated protein kinase (MAPK) pathways [45]. The exact mechanisms by which IL-9 contributes to the pathophysiology of endometriosis, whether directly or via stimulating other pro-inflammatory mediators, remain poorly understood [45]. Replication of our findings and additional mechanistic investigations are encouraged to determine whether modulating circulating IL-9 levels may offer tangible benefits for the treatment and management of endometriosis.

Finally, we found that IL-2 was causally associated with endometriosis via the WMe method with an odds ratio of 1.18. IL-2 is a pleiotropic cytokine with a variety of diverse functions and immunomodulatory actions, including driving T-cell growth and proliferation, augmenting natural killer cell cytolytic activity, and mediating activation-induced cell death, among others [48,49]. During an inflammatory response, IL-2 exhibits various and often opposing functions; thus, it has a highly complex role in the pathogenesis of systemic inflammation and inflammatory diseases more broadly [49]. Deficiency of IL-2 and its associated receptor has been associated with the development of inflammatory disorders in early rodent models [50,51]. However, a recent study reported that IL-2 levels were significantly lower in serum but higher in peritoneal fluid in women with varying stages of endometriosis compared with control women [52]. It is important to note that we did not find statistically significant associations for IL-2 via our primary IVW analysis and that the IL-2 analysis violated key assumptions of MR (with significant heterogeneity and single SNP correlations). These findings should therefore be interpreted with caution, pending replication.

### 4.3. Strengths and Limitations

The present study provides a comprehensive assessment of causal associations between serum inflammatory markers and the most common reproductive conditions, PCOS and endometriosis. Due to the robustness of MR in making causal inferences and the multiple models and analyses employed, the outcomes of this study are: (1) not susceptible to reverse causality, as demonstrated by the analyses of horizontal pleiotropy; (2) not affected by confounding factors due to Mendel’s second law of independent assortment; and (3) measured with high precision, reducing the potential for regression dilution bias, that is, the tendency of errors in the independent variable to skew linear regression towards zero [44,45].

However, some limitations are acknowledged. First, we utilised linear MR, which assumes that the exposures are linearly related to the outcome. This is not always the case, as non-linear associations have been in relation to inflammatory markers such as CRP [53]. With the advent of novel non-linear and factorial MR methods, non-linear causal associations using larger datasets should be assessed to further strengthen the evidence. However, there are controversies and retractions of non-linear MR studies [54], owing to its propensity to trigger collider bias (a distortion that modifies an association between an exposure and outcome during stratification), which could lead to spurious associations [55]. The extent to which the collider bias materially affects the effect estimate remains unknown [55]; hence, statistical rigor may be better achieved with well-established linear MR methods, until these concerns are resolved. A second limitation pertains to the IL-2 analysis, which was only significantly associated with endometriosis via the WMe method but not when using the primary IVW analysis. The WMe method yields asymptotically consistent estimates of causal effects if over 50% of the independent variables are valid and adhere to the exclusion restriction criterion; however, this assumption cannot be verified [24]. As noted above, given the significant heterogeneity and single SNP results in the IL-2 analysis, the reported association between IL-2 and endometriosis should be interpreted with caution. Third, the data used in this study were sourced solely from Finnish populations, and, although presenting the largest sample sizes with the required numbers of SNPs, there is limited generalisability to other geographic or ethnic groups. Future research should prioritise the inclusion of multi-ethnic cohorts to ensure broader applicability of findings and to explore potential population-specific genetic variations that may influence the observed associations. Fourth, despite having a sufficient number of SNPs to conduct the analysis, these were relatively limited for each exposure, potentially constraining the statistical power for detecting certain associations [56]. As repositories expand, further studies will be needed with a greater number of SNPs, a broader range of populations, and the application of non-linear MR techniques to verify the causal relationships between these markers and reproductive conditions. Fifth, PCOS prevalence appeared to be under-detected in this cohort (~0.5%) compared to its typical prevalence (10–13%) [2]. This suggests that the outcome used here reflects more severe cases of PCOS, whereas less severe cases may have been mistakenly attributed to controls. However, one would expect this to limit or mask the observed differences between cases and controls; yet, our findings reached statistical significance, suggesting that the causal associations reported here are robust. Lastly, while MCP-1/CCL2 and IL-9 are proposed as potential biomarkers for PCOS and endometriosis, respectively, supported by pre-clinical and observational data, their specificity in real-world clinical applications remains uncertain. Additional evidence is required to evaluate their reliability for use in clinical practice. Moreover, the cross-sectional nature of the GWAS data utilised in this study precludes assessments of temporal variations in inflammatory markers relative to disease progression. Longitudinal studies are warranted to clarify the dynamic relationship between inflammation and these conditions over time and potentially enhance the clinical applicability of these biomarkers.

## 5. Conclusions

Our findings provide novel evidence of circulating MCP-1/CCL2 being causally positively associated with PCOS. Elevated levels of IL-9 were also causally associated with endometriosis, while a potential positive association was detected for IL-2 with endometriosis, albeit using less reliable measures. Our results converge with previous pre-clinical and observational studies, strengthening evidence of causal associations and offering deeper insights into the critical, but complex, functions of these inflammatory markers as potential therapeutic targets for reproductive disorders. However, further validation through mechanistic and prospective studies is essential to clarify the specific functions and therapeutic potential of these markers and to determine their relevance in clinical practice.

## Figures and Tables

**Table 1:** Data retrieved from the IEU Open GWAS project to be included as exposures (inflammatory markers) and outcomes (inflammatory conditions; PCOS and endometriosis) in two-sample Mendelian randomisation analyses.

Exposure/s and Outcome/s	GWAS ID	Consortium	*n*, Participants	*n*, SNPs	Reference
Exposures: Blood-based inflammatory markers					
IL-1β	ebi-a-GCST004448	N/A	3309	9,983,642	[17]
IL-2	ebi-a-GCST004455	N/A	3475	9,512,914	[17]
IL-4	ebi-a-GCST004453	N/A	8124	9,786,064	[17]
IL-5	ebi-a-GCST004452	N/A	3364	9,450,731	[17]
IL-6	ebi-a-GCST004446	N/A	8189	9,790,590	[17]
IL-7	ebi-a-GCST004451	N/A	3409	9,692,306	[17]
IL-8	ebi-a-GCST004445	N/A	3526	9,517,348	[17]
IL-9	ebi-a-GCST004450	N/A	3634	9,567,876	[17]
IL-10	ebi-a-GCST004444	N/A	7681	9,793,415	[17]
IL-12	ebi-a-GCST004439	N/A	8270	9,799,886	[17]
IL-13	ebi-a-GCST004443	N/A	3557	9,539,073	[17]
IL-16	ebi-a-GCST004430	N/A	3483	9,551,485	[17]
IL-17	ebi-a-GCST004442	N/A	7760	9,786,653	[17]
IL-18	ebi-a-GCST004441	N/A	3636	9,785,222	[17]
MCP-1/CCL2	ebi-a-GCST004438	N/A	8293	9,801,908	[17]
CRP	ebi-a-GCST000965	N/A	15,912	30,628,777	[16]
Interferon-γ	ebi-a-GCST004456	N/A	7701	9,785,363	[17]
TNF-α	ebi-a-GCST004426	N/A	3454	9,500,449	[17]
Outcomes: Female infertility and medical assistance for female infertility					
Polycystic ovary syndrome	Finn-b-E4_PCOS	FinnGen	118,228 (642 cases and 118,228 controls)	16,379,676	N/A
Endometriosis	Finn-b-N14_ENDOMETRIOSIS	FinnGen	77,257 (8288 cases and 68,969 controls)	16,377,306	N/A

Abbreviations: CRP, c-reactive protein; GWAS, genome-wide association studies; IL, interleukin; MCP/CCL2, monocyte chemoattractant protein-1/C-C motif chemokine ligand 2; N/A, not applicable; n, sample size; PMID, pub-med identification; SNPs, single nucleotide polymorphisms; TNF-α, tumour necrosis factor-α.

**Table 2:** Selected results from two-sample Mendelian randomisation analyses of causal associations between inflammatory markers and female infertility.

Inflammatory Marker	Method	No. of SNPs	β (SE)	95% CI of β	*p*-Value	OR (95% CI)
Outcome: Polycystic ovary syndrome (finn-b-E4-PCOS)						
MCP-1/CCL2	**IVW**	**13**	**0.39 (0.15)**	**0.10, 0.69**	**0.0097**	**1.48 (1.10, 2.00)**
	MRE	13	0.48 (0.38)	−0.27, 1.23	0.2362	1.61 (0.76, 3.41)
	WMe	13	0.38 (0.21)	−0.02, 0.80	0.0583	1.47 (0.99, 2.19)
	WMo	13	0.39 (0.24)	−0.08, 0.85	0.0958	1.47 (0.97, 2.23)
	SMo	13	0.46 (0.30)	−0.14, 1.06	0.2033	1.58 (0.81, 3.09)
Outcome: Endometriosis (finn-b-E4_ENDOMETRIOSIS)						
IL-2	IVW	5	0.08 (0.10)	−0.12, 0.28	0.4403	1.08 (0.88, 1.32)
	MRE	5	0.29 (0.15)	−0.01, 0.59	0.1505	1.34 (0.99, 1.81)
	**WMe**	**5**	**0.16 (0.08)**	**0.01, 0.31**	**0.0352**	**1.18 (1.01, 1.37)**
	WMo	5	0.17 (0.08)	0.01, 0.33	0.1023	1.19 (1.01, 1.40)
	SMo	5	0.18 (0.08)	0.02, 0.35	0.0947	1.20 (1.02, 1.42)
IL-9	**IVW**	**6**	**0.14 (0.06)**	**0.02, 0.26**	**0.0277**	**1.15 (1.02, 1.30)**
	MRE	6	0.26 (0.15)	−0.02, 0.55	0.1476	1.30 (0.98, 1.73)
	WMe	6	0.15 (0.08)	−0.01, 0.32	0.0724	1.16 (0.99, 1.37)
	WMo	6	0.14 (0.10)	−0.06, 0.34	0.2243	1.15 (0.94, 1.41)
	SMo	6	0.16 (0.11)	−0.05, 0.38	0.1934	1.18 (0.95, 1.46)

Abbreviations: β, beta coefficient; CI, confidence interval; IL, interleukin; IVW, Inverse variance weighted; MCP-1/CCL-2, monocyte chemoattractant protein-1/C-C motif ligand 2; MRE, Mendelian Randomisation-Egger; OR, odds ratio; SE, standard error; SNPs, single nucleotide polymorphisms; WMe, weighted median; WMo, weighted mode. Bold figures denote a statistically significant *p*-value.

**Table 3:** Heterogeneity statistics of two-sample Mendelian randomisation analyses for significant inflammatory exposures and relevant outcomes and female infertility.

Inflammatory Marker	Method	Q-Value	Degrees of Freedom	*p*-Value
Outcome: female infertility associated with anovulation (finn-b-N14_FIANOV)				
MCP-1/CCL2	IVW	7.26	12	0.8399
	MRE	7.20	11	0.7825
Outcome: Endometriosis (finn-b-E4_ENDOMETRIOSIS)				
IL-2	IVW	13.42	4	0.0094
	MRE	6.88	3	0.0757
IL-9	IVW	2.63	5	0.7566
	MRE	1.75	4	0.7809

Abbreviations: IL, interleukin; IVW, inverse variance weighted; MCP-1/CCL-2, monocyte chemoattractant protein-1/C-C motif ligand 2; MRE, Mendelian Randomisation-Egger.

**Table 4:** Horizontal pleiotropy statistics of two-sample Mendelian randomisation analyses for significant inflammatory exposures and female infertility.

Inflammatory Marker	Egger Intercept	Standard Error	*p*-Value
Outcome: Polycystic ovary syndrome (finn-b-E4_PCOS)			
MCP-1/CCL2	−0.01	0.06	0.8124
Outcome: Endometriosis (finn-b-E4_ENDOMETRIOSIS)			
IL-2	−0.05	0.03	0.1901
IL-9	−0.03	0.03	0.4019

Abbreviations: IL, interleukin; MCP-1/CCL-2, monocyte chemoattractant protein-1/C-C motif ligand 2.

## Data Availability

The data underlying this article are publicly available as part of the Integrative Epidemiological Unit (IEU) Open Access GWAS project and FINNGEN datasets, available at https://gwas.mrcieu.ac.uk/ and https://finngen.gitbook.io/documentation/ (accessed on 15 February 2024), respectively.

## Supplementary Materials

The following supporting information can be downloaded at: https://www.mdpi.com/article/10.3390/biom14121544/s1, Table S1: Results from two-sample Mendelian Randomisation analyses of associations between inflammatory markers and inflammatory conditions (Polycystic ovary syndrome and Endometriosis); Supplementary Data File S1: Single SNP analyses; Supplementary Data File S2: Leave-one-out analyses; Supplementary Data File S3: Data harmonisation with F-statistics; Table S2: STROBE-MR Checklist [14,15].

## References

[1] Carson S.A., Kallen A.N. (2021). Diagnosis and Management of Infertility: A Review. J. Am. Med. Assoc..

[2] Teede H.J., Tay C.T., Laven J., Dokras A., Moran L.J., Piltonen T.T., Costello M.F., Boivin J., Redman L.M., Boyle J.A. (2023). Recommendations from the 2023 International Evidence-based Guideline for the Assessment and Management of Polycystic Ovary Syndrome. Fertil. Steril..

[3] Giudice L.C., Kao L.C. (2004). Endometriosis. Lancet.

[4] Burney R.O., Giudice L.C. (2012). Pathogenesis and pathophysiology of endometriosis. Fertil. Steril..

[5] Rudnicka E., Suchta K., Grymowicz M., Calik-Ksepka A., Smolarczyk K., Duszewska A.M., Smolarczyk R., Meczekalski B. (2021). Chronic Low Grade Inflammation in Pathogenesis of PCOS. Int. J. Mol. Sci..

[6] Orisaka M., Mizutani T., Miyazaki Y., Shirafuji A., Tamamura C., Fujita M., Tsuyoshi H., Yoshida Y. (2023). Chronic low-grade inflammation and ovarian dysfunction in women with polycystic ovarian syndrome, endometriosis, and aging. Front. Endocrinol..

[7] Shorakae S., Ranasinha S., Abell S., Lambert G., Lambert E., de Courten B., Teede H. (2018). Inter-related effects of insulin resistance, hyperandrogenism, sympathetic dysfunction and chronic inflammation in PCOS. Clin. Endocrinol..

[8] Velez L.M., Seldin M., Motta A.B. (2021). Inflammation and reproductive function in women with polycystic ovary syndrome†. Biol. Reprod..

[9] Parasar P., Ozcan P., Terry K.L. (2017). Endometriosis: Epidemiology, Diagnosis and Clinical Management. Curr. Obstet. Gynecol. Rep..

[10] Vannuccini S., Clifton V.L., Fraser I.S., Taylor H.S., Critchley H., Giudice L.C., Petraglia F. (2016). Infertility and reproductive disorders: Impact of hormonal and inflammatory mechanisms on pregnancy outcome. Hum. Reprod. Update.

[11] Escobar-Morreale H.F., Luque-Ramírez M., González F. (2011). Circulating inflammatory markers in polycystic ovary syndrome: A systematic review and metaanalysis. Fertil. Steril..

[12] De Silva K., Demmer R.T., Jönsson D., Mousa A., Teede H., Forbes A., Enticott J. (2022). Causality of anthropometric markers associated with polycystic ovarian syndrome: Findings of a Mendelian randomization study. PLoS ONE.

[13] Davey Smith G., Holmes M.V., Davies N.M., Ebrahim S. (2020). Mendel’s laws, Mendelian randomization and causal inference in observational data: Substantive and nomenclatural issues. Eur. J. Epidemiol..

[14] Skrivankova V.W., Richmond R.C., Woolf B.A.R., Yarmolinsky J., Davies N.M., Swanson S.A., VanderWeele T.J., Higgins J.P.T., Timpson N.J., Dimou N. (2021). Strengthening the Reporting of Observational Studies in Epidemiology Using Mendelian Randomization: The STROBE-MR Statement. J. Am. Med. Assoc..

[15] Skrivankova V.W., Richmond R.C., Woolf B.A.R., Davies N.M., Swanson S.A., VanderWeele T.J., Timpson N.J., Higgins J.P.T., Dimou N., Langenberg C. (2021). Strengthening the reporting of observational studies in epidemiology using mendelian randomisation (STROBE-MR): Explanation and elaboration. Br. Med. J..

[16] Dehghan A., Dupuis J., Barbalic M., Bis J.C., Eiriksdottir G., Lu C., Pellikka N., Wallaschofski H., Kettunen J., Henneman P. (2011). Meta-analysis of genome-wide association studies in >80 000 subjects identifies multiple loci for C-reactive protein levels. Circulation.

[17] Ahola-Olli A.V., Würtz P., Havulinna A.S., Aalto K., Pitkänen N., Lehtimäki T., Kähönen M., Lyytikäinen L.P., Raitoharju E., Seppälä I. (2017). Genome-wide Association Study Identifies 27 Loci Influencing Concentrations of Circulating Cytokines and Growth Factors. Am. J. Hum. Genet..

[18] Mackay T.F.C., Anholt R.R.H. (2022). Gregor Mendel’s legacy in quantitative genetics. PLoS Biol..

[19] Gagliano Taliun S.A., Evans D.M. (2021). Ten simple rules for conducting a mendelian randomization study. PLoS Comput. Biol..

[20] Zhang N., Liao Y., Zhao H., Chen T., Jia F., Yu Y., Zhu S., Wang C., Zhang W., Liu X. (2023). Polycystic ovary syndrome and 25-hydroxyvitamin D: A bidirectional two-sample Mendelian randomization study. Front. Endocrinol..

[21] Chen Z., Boehnke M., Wen X., Mukherjee B. (2021). Revisiting the genome-wide significance threshold for common variant GWAS. G3.

[22] Burton P.R., Clayton D.G., Cardon L.R., Craddock N., Deloukas P., Duncanson A., Kwiatkowski D.P., McCarthy M.I., Ouwehand W.H., Samani N.J. (2007). Genome-wide association study of 14,000 cases of seven common diseases and 3000 shared controls. Nature.

[23] Hammond R.K., Pahl M.C., Su C., Cousminer D.L., Leonard M.E., Lu S., Doege C.A., Wagley Y., Hodge K.M., Lasconi C. (2021). Biological constraints on GWAS SNPs at suggestive significance thresholds reveal additional BMI loci. elife.

[24] Burgess S., Davey Smith G., Davies N., Dudbridge F., Gill D., Glymour M., Hartwig F., Holmes M., Minelli C., Relton C. (2020). Guidelines for performing Mendelian randomization investigations [version 2; peer review: 2 approved]. Wellcome Open Res..

[25] Hartwig F.P., Davey Smith G., Bowden J. (2017). Robust inference in summary data Mendelian randomization via the zero modal pleiotropy assumption. Int. J. Epidemiol..

[26] Lin Z., Shi J.L., Chen M., Zheng Z.M., Li M.Q., Shao J. (2022). CCL2: An important cytokine in normal and pathological pregnancies: A review. Front. Immunol..

[27] Bouet P.E., Chao de la Barca J.M., Boucret L., Descamps P., Legendre G., Hachem H.E., Blanchard S., Jeannin P., Reynier P., May-Panloup P. (2020). Elevated Levels of Monocyte Chemotactic Protein-1 in the Follicular Fluid Reveals Different Populations among Women with Severe Endometriosis. J. Clin. Med..

[28] Singh S., Anshita D., Ravichandiran V. (2021). MCP-1: Function, regulation, and involvement in disease. Int. Immunopharmacol..

[29] Wu Z., Fang L., Li Y., Yan Y., Thakur A., Cheng J.C., Sun Y.P. (2021). Association of circulating monocyte chemoattractant protein-1 levels with polycystic ovary syndrome: A meta-analysis. Am. J. Reprod. Immunol..

[30] González F., Rote N.S., Minium J., Kirwan J.P. (2009). Evidence of proatherogenic inflammation in polycystic ovary syndrome. Metab. Clin. Exp..

[31] Atabekoglu C.S., Sönmezer M., Özmen B., Yarcı A., Akbıyık F., Taşçı T., Aytaç R. (2011). Increased monocyte chemoattractant protein-1 levels indicating early vascular damage in lean young PCOS patients. Fertil. Steril..

[32] Hu W., Qiao J., Yang Y., Wang L., Li R. (2011). Elevated C-reactive protein and monocyte chemoattractant protein-1 in patients with polycystic ovary syndrome. Eur. J. Obstet. Gynecol. Reprod. Biol..

[33] Li L., Ryoo J.E., Lee K.J., Choi B.C., Baek K.H. (2015). Genetic variation in the Mcp-1 gene promoter associated with the risk of polycystic ovary syndrome. PLoS ONE.

[34] Glintborg D., Andersen M., Richelsen B., Bruun J.M. (2009). Plasma monocyte chemoattractant protein-1 (MCP-1) and macrophage inflammatory protein-1α are increased in patients with polycystic ovary syndrome (PCOS) and associated with adiposity, but unaffected by pioglitazone treatment. Clin. Endocrinol..

[35] Gateva A., Kamenov Z., Tsakova A. (2013). MCP-1 and fetuin A levels in patients with PCOS and/or obesity before and after metformin treatment. Cent. Eur. J. Med..

[36] Ciaraldi T.P., Aroda V., Mudaliar S.R., Henry R.R. (2013). Inflammatory cytokines and chemokines, skeletal muscle and polycystic ovary syndrome: Effects of pioglitazone and metformin treatment. Metabolism.

[37] Hu W.H., Qiao J., Li M.Z. (2007). Association of monocyte chemoattractant protein-1 and the clinical characteristics of polycystic ovary syndrome: Analysis of 65 cases. Zhonghua Yi Xue Za Zhi.

[38] Kamei N., Tobe K., Suzuki R., Ohsugi M., Watanabe T., Kubota N., Ohtsuka-Kowatari N., Kumagai K., Sakamoto K., Kobayashi M. (2006). Overexpression of Monocyte Chemoattractant Protein-1 in Adipose Tissues Causes Macrophage Recruitment and Insulin Resistance. J. Biol. Chem..

[39] Evers-van Gogh I.J.A., Oteng A.-B., Alex S., Hamers N., Catoire M., Stienstra R., Kalkhoven E., Kersten S. (2016). Muscle-specific inflammation induced by MCP-1 overexpression does not affect whole-body insulin sensitivity in mice. Diabetologia.

[40] Dahm-Kähler P., Runesson E., Lind A.K., Brännström M. (2006). Monocyte chemotactic protein-1 in the follicle of the menstrual and IVF cycle. Mol. Hum. Reprod..

[41] Balen A.H., Rutherford A.J. (2007). Managing anovulatory infertility and polycystic ovary syndrome. Br. Med. J..

[42] Di Christine Oliveira Y.L., de Oliveira Y.L.M., Cirilo T.M., Fujiwara R.T., Bueno L.L., Dolabella S.S. (2023). Inflammatory Profile of Th9 Cells and Their Protective Potential in Helminth Infections. Immunology.

[43] Temann U.A., Laouar Y., Eynon E.E., Homer R., Flavell R.A. (2007). IL9 leads to airway inflammation by inducing IL13 expression in airway epithelial cells. Int. Immunol..

[44] Chakraborty S., Kubatzky K.F., Mitra D.K. (2019). An Update on Interleukin-9: From Its Cellular Source and Signal Transduction to Its Role in Immunopathogenesis. Int. J. Mol. Sci..

[45] Tarumi Y., Mori T., Okimura H., Maeda E., Tanaka Y., Kataoka H., Ito F., Koshiba A., Kusuki I., Kitawaki J. (2021). Interleukin-9 produced by helper T cells stimulates interleukin-8 expression in endometriosis. Am. J. Reprod. Immunol..

[46] Jørgensen H., Hill A.S., Beste M.T., Kumar M.P., Chiswick E., Fedorcsak P., Isaacson K.B., Lauffenburger D.A., Griffith L.G., Qvigstad E. (2017). Peritoneal fluid cytokines related to endometriosis in patients evaluated for infertility. Fertil. Steril..

[47] Zanotta N., Monasta L., Skerk K., Luppi S., Martinelli M., Ricci G., Comar M. (2019). Cervico-vaginal secretion cytokine profile: A non-invasive approach to study the endometrial receptivity in IVF cycles. Am. J. Reprod. Immunol..

[48] Liao W., Lin J.X., Leonard W.J. (2011). IL-2 family cytokines: New insights into the complex roles of IL-2 as a broad regulator of T helper cell differentiation. Curr. Opin. Immunol..

[49] Hoyer K.K., Dooms H., Barron L., Abbas A.K. (2008). Interleukin-2 in the development and control of inflammatory disease. Immunol. Rev..

[50] Sadlack B., Löhler J., Schorle H., Klebb G., Haber H., Sickel E., Noelle R.J., Horak I. (1995). Generalized autoimmune disease in interleukin-2-deficient mice is triggered by an uncontrolled activation and proliferation of CD4+ T cells. Eur. J. Immunol..

[51] Sadlack B., Merz H., Schorle H., Schimpl A., Feller A.C., Horak I. (1993). Ulcerative colitis-like disease in mice with a disrupted interleukin-2 gene. Cell.

[52] Fan Y.-Y., Chen H.-Y., Chen W., Liu Y.-N., Fu Y., Wang L.-N. (2018). Expression of inflammatory cytokines in serum and peritoneal fluid from patients with different stages of endometriosis. Gynecol. Endocrinol..

[53] Zhou A., Hyppönen E. (2023). Vitamin D deficiency and C-reactive protein: A bidirectional Mendelian randomization study. Int. J. Epidemiol..

[54] Sofianopoulou E., Kaptoge S.K., Afzal S., Jiang T., Gill D., Gundersen T.E., Bolton T.R., Allara E., Arnold M.G., Mason A.M. (2021). RETRACTED: Estimating dose-response relationships for vitamin D with coronary heart disease, stroke, and all-cause mortality: Observational and Mendelian randomisation analyses. Lancet Diabetes Endocrinol..

[55] Wade K.H., Hamilton F.W., Carslake D., Sattar N., Davey Smith G., Timpson N.J. (2023). Challenges in undertaking nonlinear Mendelian randomization. Obesity.

[56] Burgess S., Davey Smith G., Davies N.M., Dudbridge F., Gill D., Glymour M.M., Hartwig F.P., Kutalik Z., Holmes M.V., Minelli C. (2019). Guidelines for performing Mendelian randomization investigations: Update for summer 2023. Wellcome Open Res..

