# Supplementary material for: Exploring Causal Associations Between Serum Inflammatory Markers and Female Reproductive Disorders: A Mendelian Randomisation Study
Source: Biomolecules. 2024 Dec 2;14(12):1544. doi: 10.3390/biom14121544 (PMC11674023; doi:10.3390/biom14121544)
Supplement: Supplementary file 1 [file biomolecules-14-01544-s001.zip › Figures 1-3 for main manuscript.pdf]

A)

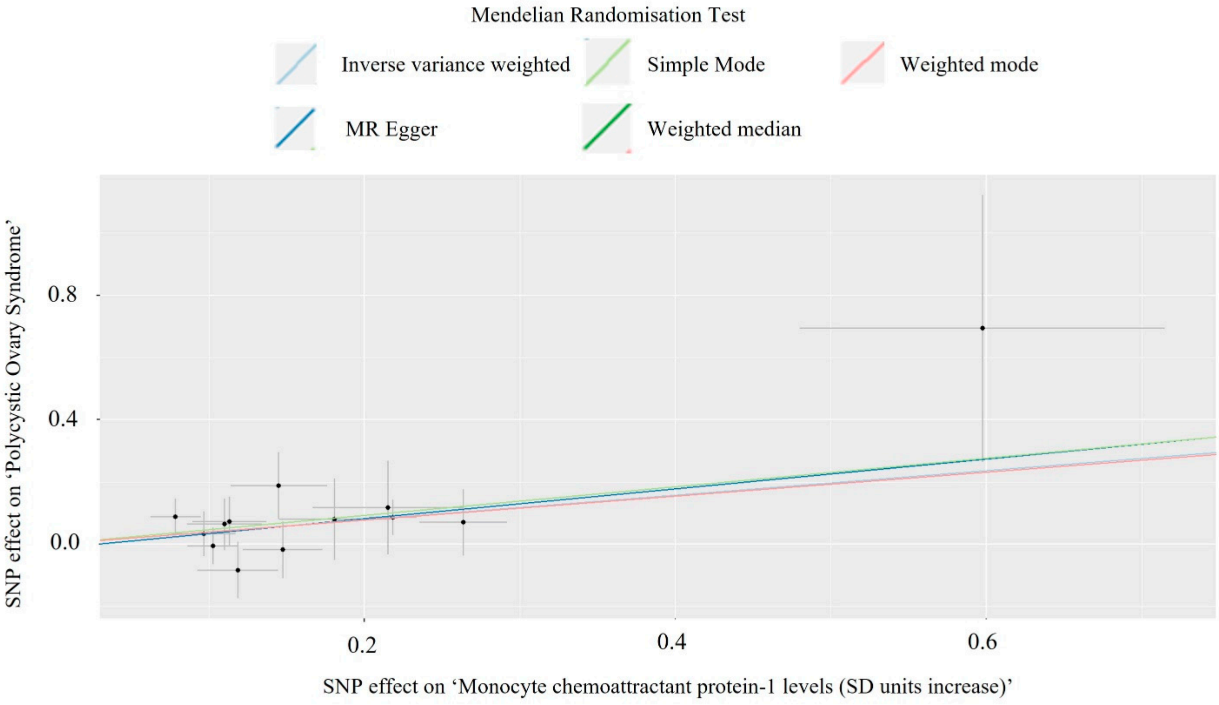

Mendelian Randomisation Test
Inverse variance weighted
Simple Mode
Weighted mode
MR Egger
Weighted median
SNP effect on 'Polycystic Ovary Syndrome'
0.8
0.4
0.0
0.2
0.4
0.6
SNP effect on 'Monocyte chemoattractant protein-1 levels (SD units increase)'

B)

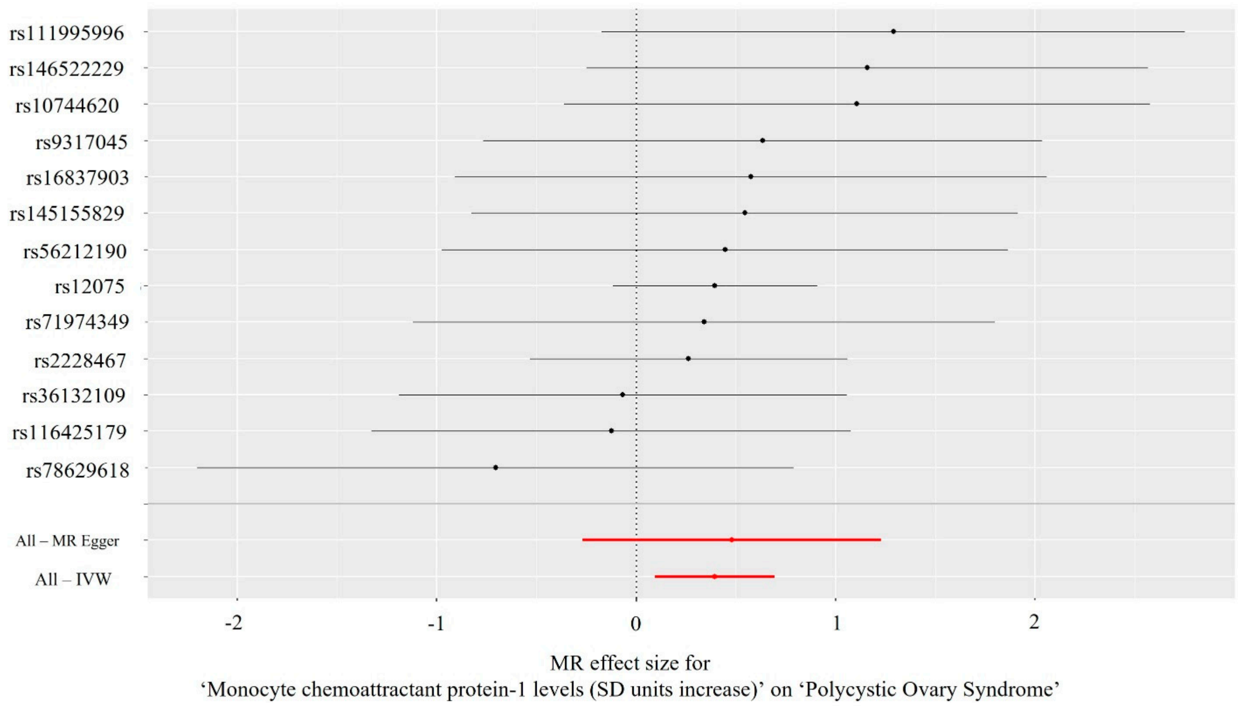

rs111995996
rs146522229
rs10744620
rs9317045
rs16837903
rs145155829
rs56212190
rs12075
rs71974349
rs2228467
rs36132109
rs116425179
rs78629618
All – MR Egger
All – IVW
-2
-1
0
1
2
MR effect size for
'Monocyte chemoattractant protein-1 levels (SD units increase)' on 'Polycystic Ovary Syndrome'

C)

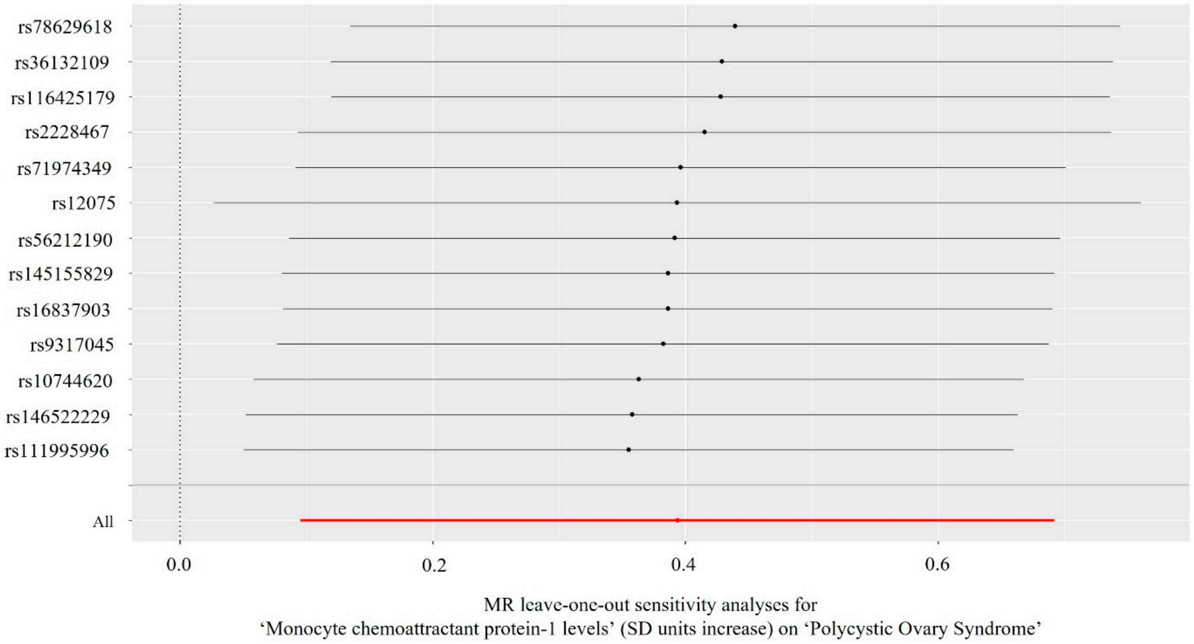

D)

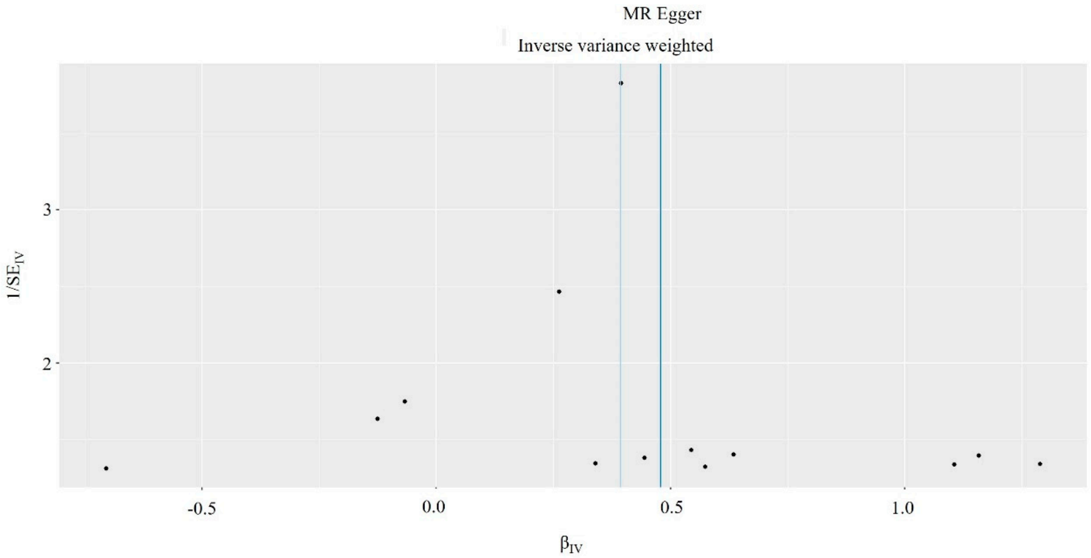

Figure 1: The relationship between **'**monocyte chemoattractant protein-1 (MCP-1/CCL2) (standard deviation units increase)' and the outcome of 'polycystic ovary syndrome', visualised by: **(A)** Scatter plot denoting the distribution of individual causal estimates, with lines indicating the trendlines of the causal estimate using each method; **(B)** Forest plot of the effects of individual single nucleotide polymorphisms (SNPs) and pooled estimates visualised via mendelian randomisation-egger (MRE) and inverse variance weighted (IVW) methods; **(C)** Leave-one-out analysis plot, where the dark points indicate the effect measure via IVW-mendelian randomisation (IVW-MR) analysis excluding the specific SNP. The red line indicates the pooled analysis encompassing all SNPs via IVW-MR; and **(D)** Funnel plot to assess heterogeneity.

A)

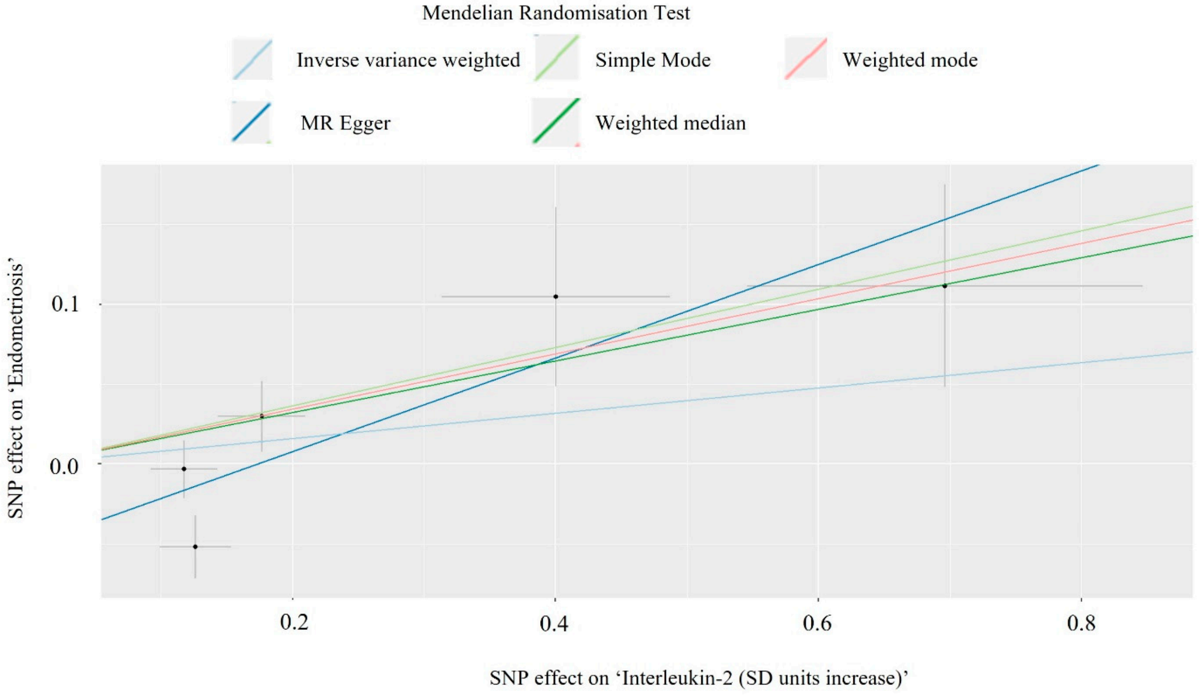

Mendelian Randomisation Test
Inverse variance weighted
Simple Mode
Weighted mode
MR Egger
Weighted median
SNP effect on 'Endometriosis'
0.1
0.0
0.2
0.4
0.6
0.8
SNP effect on 'Interleukin-2 (SD units increase)'

B)

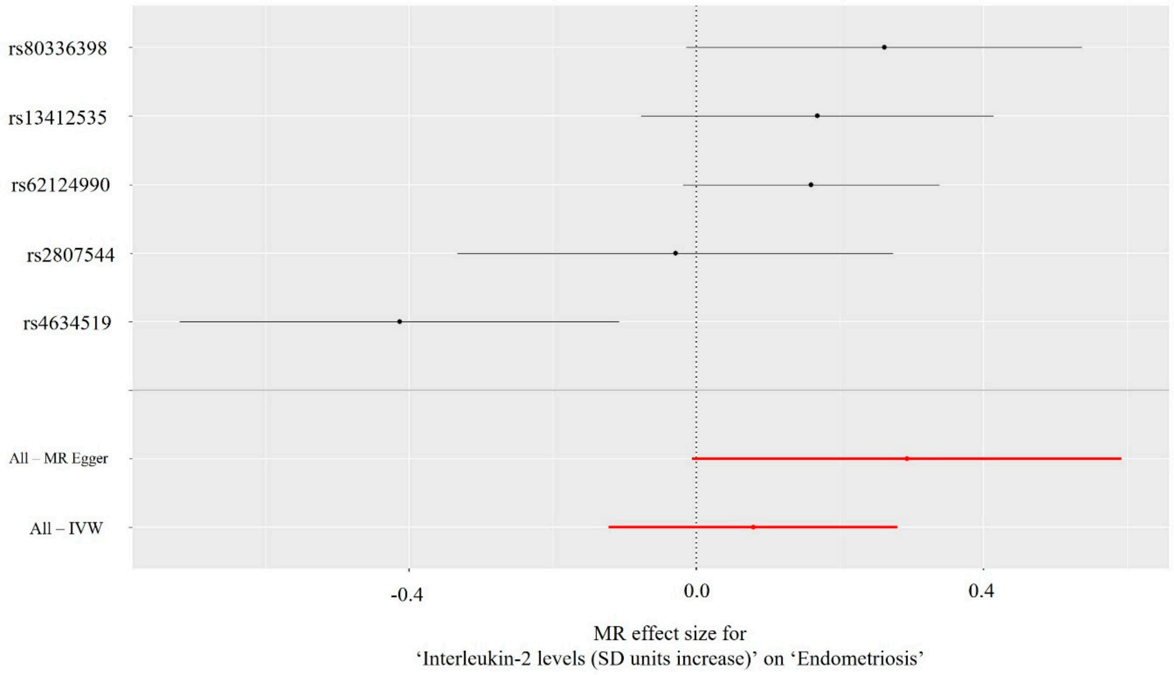

rs80336398
rs13412535
rs62124990
rs2807544
rs4634519
All – MR Egger
All – IVW
-0.4
0.0
0.4
MR effect size for
'Interleukin-2 levels (SD units increase)' on 'Endometriosis'

C)

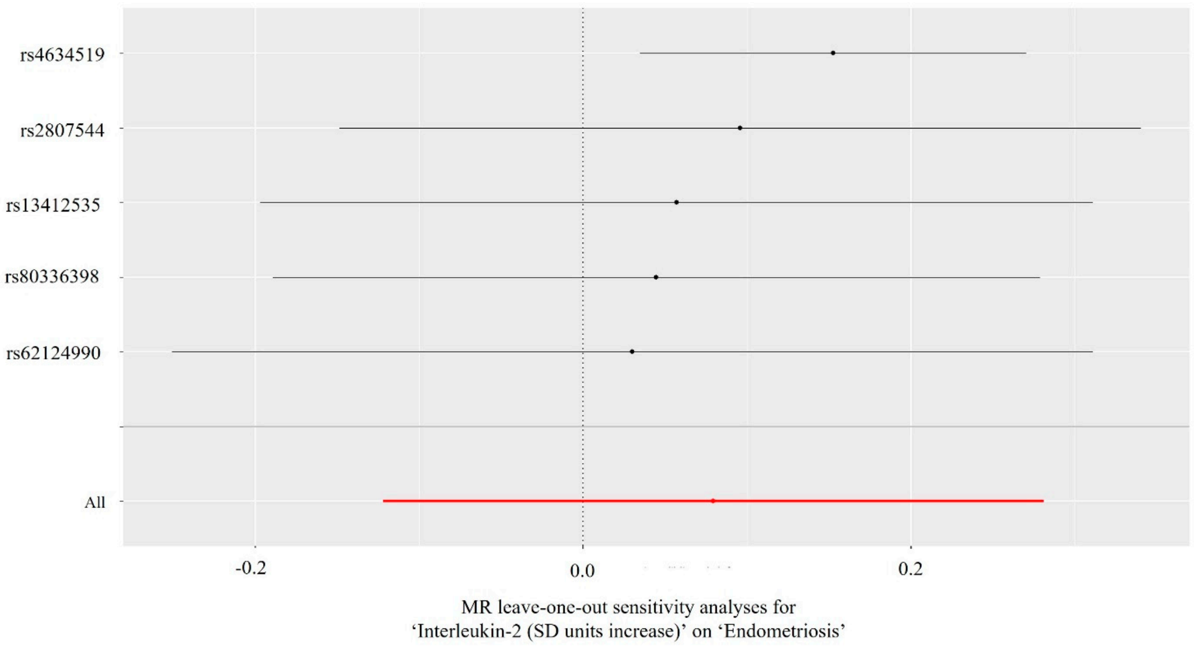

D)

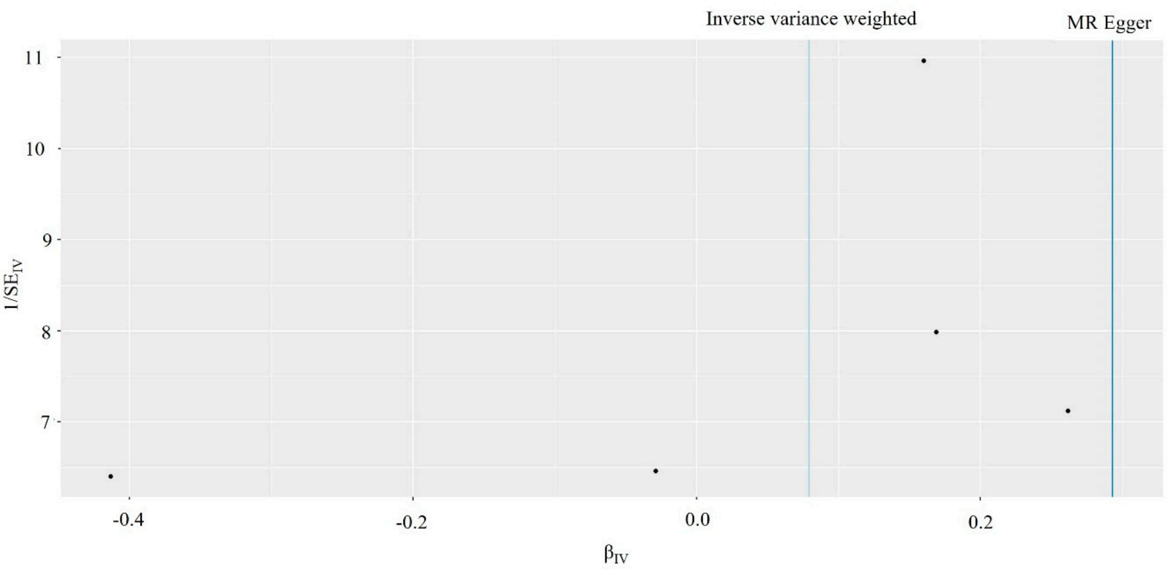

Figure 2: The relationship between 'interleukin-2 (IL-2) (standard deviation units increase)' and the outcome of 'endometriosis', visualised by: **(A)** Scatter plot denoting the distribution of individual causal estimates, with lines indicating the trendlines of the causal estimate using each method; **(B)** Forest plot of the effects of individual single nucleotide polymorphisms (SNPs) and pooled estimates visualised via mendelian randomisation-egger (MRE) and inverse variance weighted (IVW) methods; **(C)** Leave-one-out analysis plot, where the dark points indicate the effect measure via IVW-mendelian randomisation (IVW-MR) analysis excluding the specific SNP. The red line indicates the pooled analysis encompassing all SNPs via IVW-MR; and **(D)** Funnel plot to assess heterogeneity.

A)

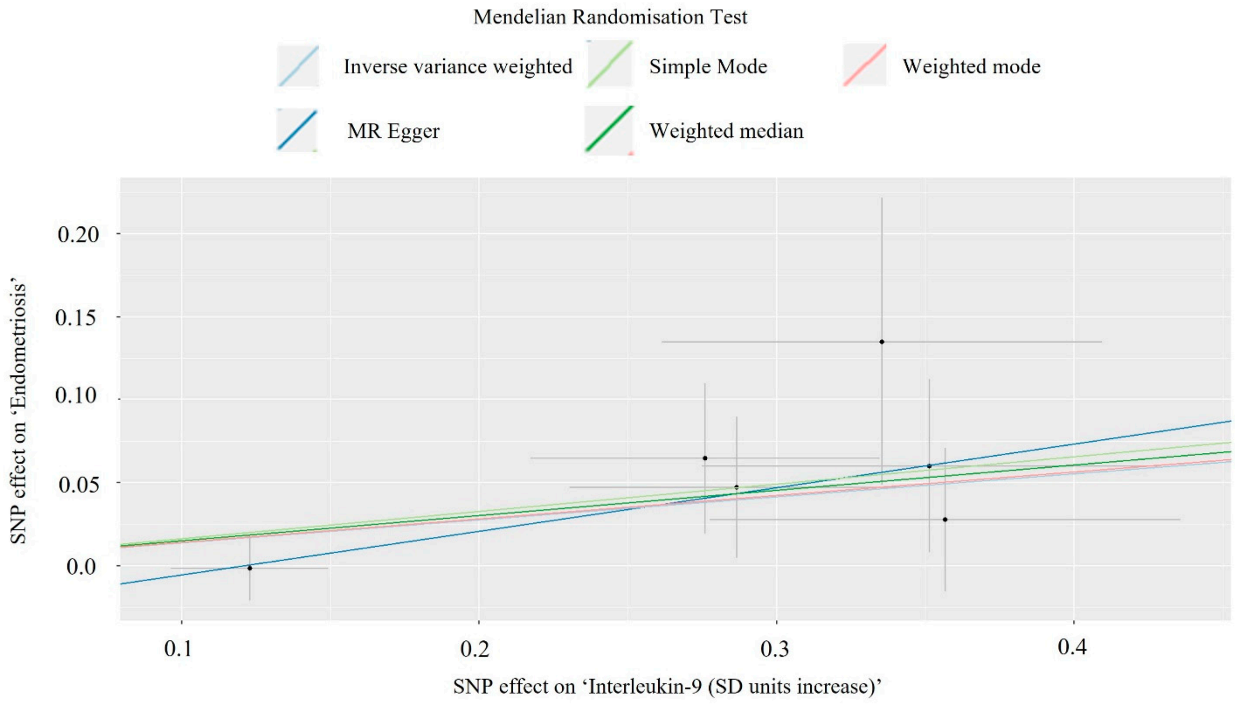
Mendelian Randomisation Test
Inverse variance weighted
Simple Mode
Weighted mode
MR Egger
Weighted median
SNP effect on 'Endometriosis'
0.20
0.15
0.10
0.05
0.0
0.1
0.2
0.3
0.4
SNP effect on 'Interleukin-9 (SD units increase)'

B)

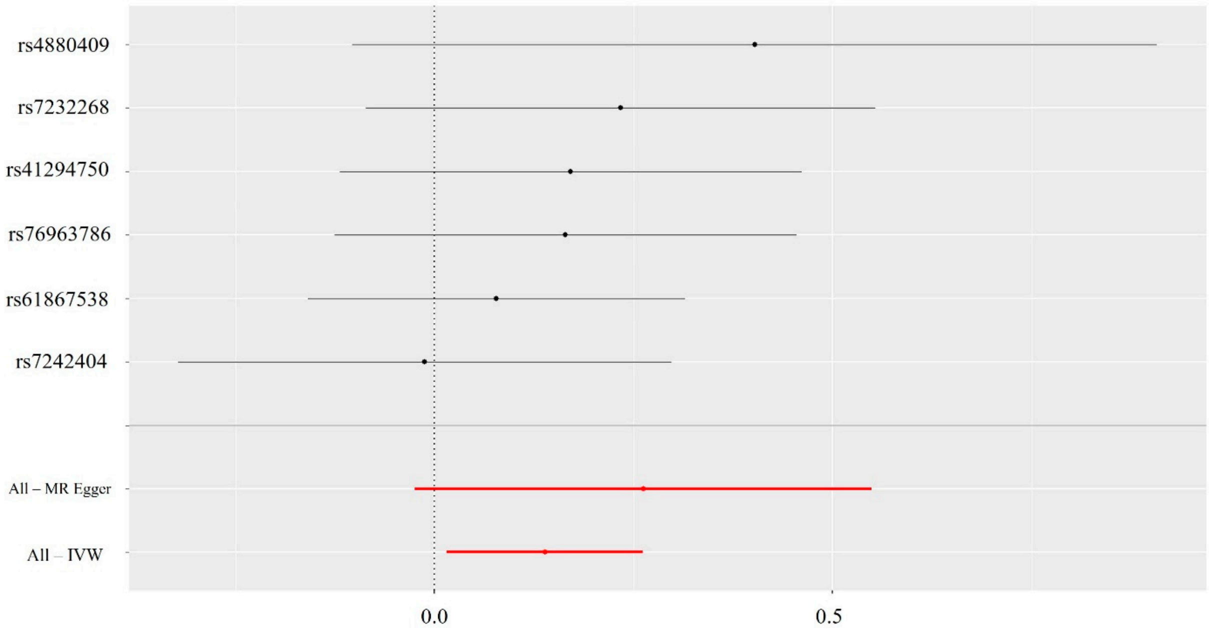
rs4880409
rs7232268
rs41294750
rs76963786
rs61867538
rs7242404
All – MR Egger
All – IVW
0.0
0.5

C)

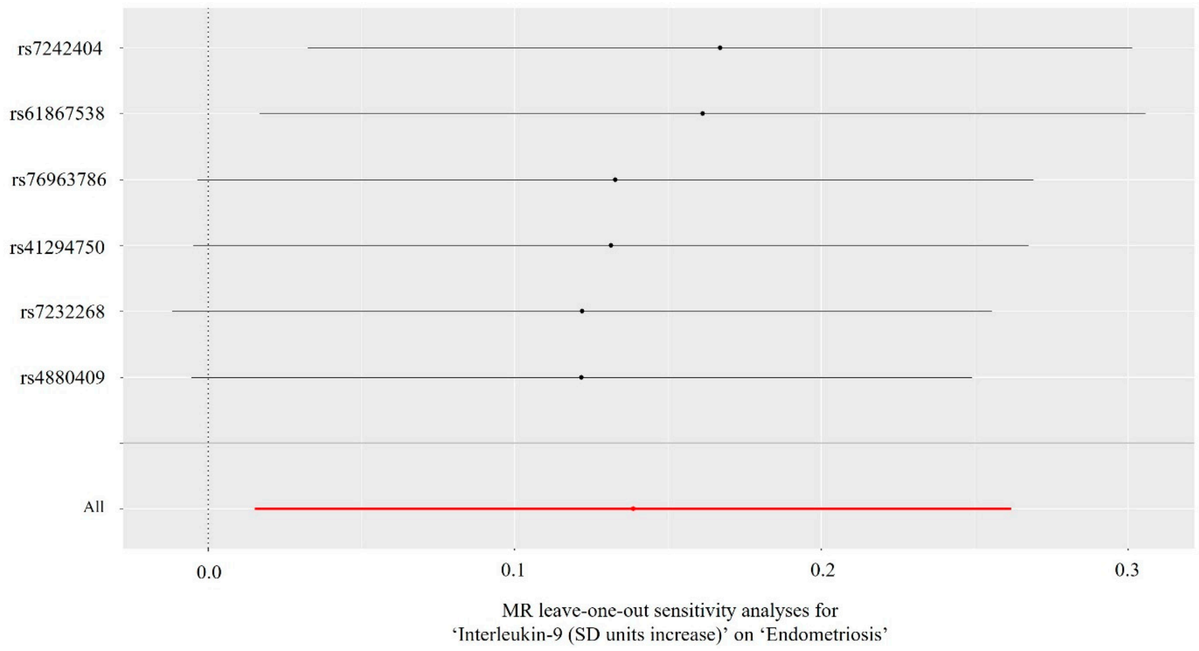

D)

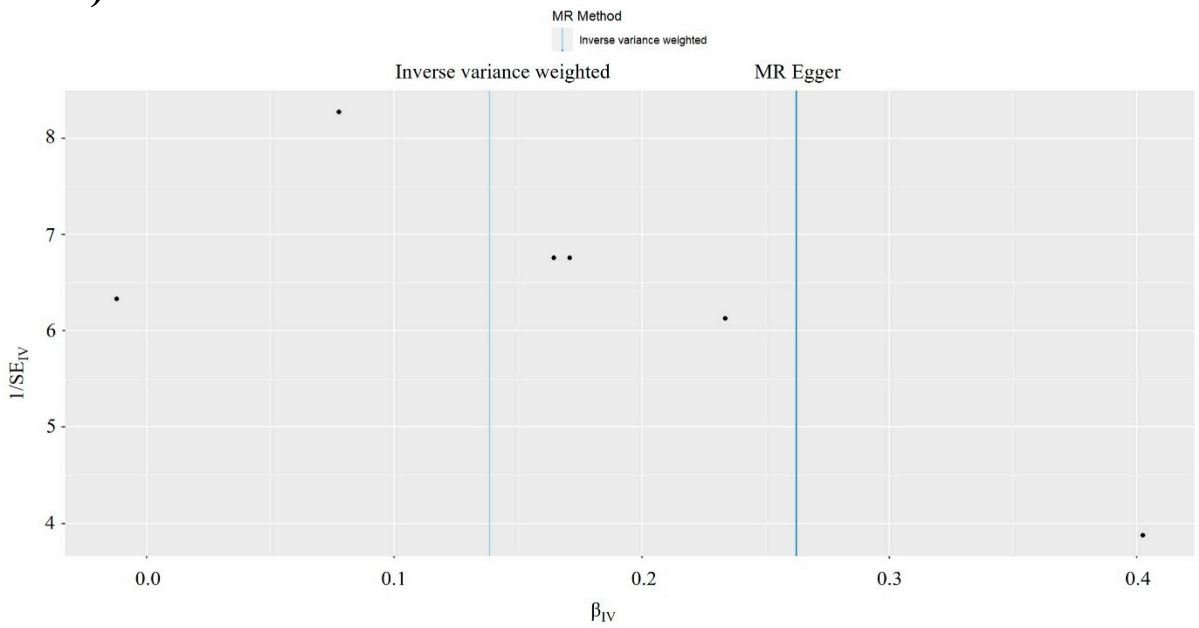

Figure 3: The relationship between 'interleukin-9 (IL-9) (standard deviation units increase)' and the outcome of 'endometriosis', visualised by: **(A)** Scatter plot denoting the distribution of individual causal estimates, with lines indicating the trendlines of the causal estimate using each method; **(B)** Forest plot of the effects of individual single nucleotide polymorphisms (SNPs) and pooled estimates visualised via mendelian randomisation-egger (MRE) and inverse variance weighted (IVW) methods; **(C)** Leave-one-out analysis plot, where the dark points indicate the effect measure via IVW-mendelian randomisation (IVW-MR) analysis excluding the specific SNP. The red line indicates the pooled analysis encompassing all SNPs via IVW-MR; and **(D)** Funnel plot to assess heterogeneity.
