# Supplementary material for: Exploring Causal Associations Between Serum Inflammatory Markers and Female Reproductive Disorders: A Mendelian Randomisation Study
Source: Biomolecules. 2024 Dec 2;14(12):1544. doi: 10.3390/biom14121544 (PMC11674023; doi:10.3390/biom14121544)
Supplement: Supplementary file 1 [file biomolecules-14-01544-s001.zip › Table S1.pdf]

Table S1. Results from two-sample Mendelian Randomisation analyses of associations between inflammatory markers and inflammatory conditions (Polycystic ovary syndrome and Endometriosis)

**Outcome:**		**Polycystic ovary syndrome**					**Endometriosis**				
**Exposure**	**SNPs**	**IVW**	**MRE**	**WMe**	**WMo**	**SMo**	**IVW**	**MRE**	**WMe**	**WMo**	**SMo**
IL-1β	3	1.10 (0.67, 1.83)	1.86 (0.27, 12.99)	1.11 (0.62, 1.98)	1.24 (0.71, 2.17)	0.70 (0.29, 1.69)	0.95 (0.81, 1.11)	1.45 (0.80, 2.64)	0.97 (0.81, 1.17)	0.98 (0.81, 1.19)	1.01 (0.79, 1.30)
IL-2	5	0.99 (0.61, 1.64)	1.03 (0.37, 2.91)	0.96 (0.59, 1.56)	1.39 (0.71, 2.72)	0.71 (0.29, 1.75)	1.08 (0.88, 1.32)	1.34 (0.99, 1.81)	**1.18 (1.01, 1.37)**	1.19 (1.01, 1.40)	1.20 (1.02, 1.42)
IL-4	5	0.67 (0.35, 1.27)	0.73 (0.18, 2.93)	0.93 (0.40, 2.16)	1.01 (0.33, 3.05)	1.01 (0.33, 3.04)	1.07 (0.82, 1.39)	0.76 (0.51, 1.12)	1.12 (0.84, 1.49)	1.14 (0.76, 1.72)	1.13 (0.73, 1.75)
IL-5	5	0.73 (0.50, 1.08)	0.60 (0.24, 1.50)	0.82 (0.51, 1.33)	0.84 (0.44, 1.59)	0.83 (0.44, 1.57)	1.08 (0.95, 1.21)	1.09 (0.82, 1.44	1.08 (0.94, 1.24)	1.09 (0.91, 1.31)	1.09 (0.91, 1.31)
IL-6	5	1.70 (0.94, 3.05)	1.67 (0.40 7.01)	1.63 (0.81, 3.31)	1.34 (0.50, 3.59)	1.25 (0.46, 3.37)	0.92 (0.72, 1.17)	0.96 (0.48, 1.90)	0.85 (0.65, 1.09)	0.75 (0.49, 1.15)	0.74 (0.51, 1.08)
IL-7	9	0.90 (0.67, 1.2)	0.85 (0.46, 1.56)	0.82 (0.56, 1.20)	0.77 (0.46, 1.29)	0.76 (0.47, 1.25)	1.02 (0.89, 1.17)	1.26 (0.99, 1.60)	1.02 (0.89, 1.18)	0.91 (0.67, 1.24)	0.95 (0.70, 1.29)
IL-8	4	0.97 (0.54, 1.75)	0.42 (0.10, 1.67)	0.89 (0.48, 1.65)	0.72 (0.27, 1.92)	0.70 (0.26, 1.91)	1.09 (0.94, 1.27)	1.24 (0.83, 1.84)	1.15 (0.97, 1.38)	1.16 (0.89, 1.51)	1.16 (0.90, 1.50)
IL-9	6	0.75 (0.47, 1.19)	1.50 (0.59, 3.83)	0.81 (0.47, 1.39)	0.79 (0.40, 1.58)	0.78 (0.34, 1.79)	**1.15 (1.02, 1.30)**	1.30 (0.98, 1.73)	1.16 (0.99, 1.37)	1.15 (0.94, 1.41)	1.18 (0.95, 1.46)
IL-10	10	0.73 (0.48, 1.12)	9.86 (0.21, 456.67)	0.79 (0.45, 1.39)	0.86 (0.37, 2.01)	0.88 (0.35, 2.18)	0.99 (0.87, 1.13)	0.72 (0.22, 2.34)	0.98 (0.83, 1.17)	0.96 (0.73, 1.25)	0.95 (0.73, 1.24)
IL-12	8	0.90 (0.48, 1.71)	1.86 (0.37, 9.33)	0.72 (0.37, 1.40)	0.71 (0.24, 2.14)	0.72 (0.22, 2.38)	0.98 (0.85, 1.14)	1.05 (0.72, 1.53)	0.96 (0.79, 1.17)	0.93 (0.70, 1.23)	0.95 (0.68, 1.31)
IL-13	7	1.00 (0.80, 1.24)	1.00 (0.66, 1.51)	0.98 (0.76, 1.26)	0.96 (0.73, 1.26)	1.26 (0.78, 2.04)	1.03 (0.96, 1.10)	1.03 (0.91, 1.17)	1.03 (0.95, 1.12)	1.03 (0.96, 1.12)	1.011 (0.88, 1.16)
IL-16	8	0.91 (0.57, 1.46)	0.89 (0.37, 2.13)	1.00 (0.72, 1.37)	1.01 (0.70, 1.45)	0.87 (0.43, 1.77)	0.94 (0.88, 1.02)	1.01 (0.89, 1.15)	0.95 (0.86, 1.04)	1.012 (0.91, 1.13)	0.87 (0.72, 1.05)
IL-17	8	1.01 (0.70, 1.53)	2.01 (0.87, 4.60)	1.05 (0.60, 1.83)	1.15 (0.55, 2.40)	1.04 (0.44, 2.46)	0.99 (0.87, 1.12)	0.98 (0.76, 1.27)	1.03 (0.87, 1.21)	1.04 (0.84, 1.28)	1.02 (0.78, 1.33)
IL-18	11	0.91 (0.73, 1.13)	0.95 (0.60, 1.51)	0.84 (0.63, 1.12)	0.85 (0.59, 1.24)	0.88 (0.55, 1.43)	1.00 (0.92, 1.09	0.98 (0.82, 1.19)	0.96 (0.87, 1.06)	0.96 (0.83, 1.11)	1.15 (0.95, 1.40)
MCP-1/ CCL2	13	**1.48 (1.10, 2.00)**	1.61 (0.76, 3.41)	1.47 (0.99, 2.19)	1.47 (0.97, 2.23)	1.58 (0.81, 3.09)	0.98 (0.90, 1.08)	1.02 (0.81, 1.29)	1.06 (0.93, 1.21)	1.05 (0.91, 1.22)	0.95 (0.75, 1.20)
CRP	17	0.76 (0.51, 1.14)	1.12 (0.60, 2.12)	0.88 (0.55, 1.40)	0.89 (0.58, 1.37)	0.63 (0.23, 1.79)	1.01 (0.90, 1.13)	0.93 (0.78, 1.12)	1.09 (0.92, 1.28)	1.10 (0.93, 1.31)	1.16 (0.84, 1.60)
Interferon-γ	5	0.97 (0.33, 2.81)	0.72 (0.08, 6.53)	1.65 (0.76, 3.57)	1.97 (0.88, 4.40)	1.91 (0.90, 4.07)	0.99 (0.83, 1.17)	0.86 (0.63, 1.17)	0.97 (0.78, 1.22)	0.97 (0.73, 1.28)	1.00 (0.74, 1.35)
TNF-α	3	0.73 (0.38, 1.39)	0.43 (0.19, 0.96)	0.59 (0.31, 1.13)	0.55 (0.26, 1.20)	0.56 (0.24, 1.30)	1.06 (0.91, 1.24)	1.11 (0.86, 1.44)	1.08 (0.89, 1.30)	1.09 (0.87, 1.36)	1.09 (0.87, 1.36)

All data are reported as odds ratios (95% confidence interval), unless otherwise specified. Bold figures denote a statistically significant association with a p-value <0.05.

Abbreviations: **CRP,** c-reactive protein; **IL**, interleukin; **IVW**, Inverse variance weighted; **MCP/CCL2,** monocyte chemoattractant protein-1/ C-C motif chemokine ligand 2; **MRE**, Mendelian Randomisation-Egger; **SNPs**, single nucleotide polymorphisms; **TNF-α**, tumour necrosis factor-α; **WMe**, weighted median; **WMo**, weighted mode.
