# Supplementary material for: Exploring Causal Associations Between Serum Inflammatory Markers and Female Reproductive Disorders: A Mendelian Randomisation Study
Source: Biomolecules. 2024 Dec 2;14(12):1544. doi: 10.3390/biom14121544 (PMC11674023; doi:10.3390/biom14121544)
Supplement: Supplementary file 1 [file biomolecules-14-01544-s001.zip › Table S2.pdf]

Table S2. STROBE-MR checklist of recommended items to address in reports of Mendelian randomization studies[1 2]

Item No.	Section		Checklist item	Location	Relevant text from manuscript
1	**TITLE and ABSTRACT**		Indicate Mendelian randomization (MR) as the study's design in the title and/or the abstract if that is a main purpose of the study	Page 1	Title: Causal associations between serum inflammatory markers and female reproductive disorders: a Mendelian Randomisation study.
	**INTRODUCTION**				
2	**Background**		Explain the scientific background and rationale for the reported study. What is the exposure? Is a potential causal relationship between exposure and outcome plausible? Justify why MR is a helpful method to address the study question	Page 3	Second paragraph of the introduction.
3	**Objectives**		State specific objectives clearly, including pre-specified causal hypotheses (if any). State that MR is a method that, under specific assumptions, intends to estimate causal effects	Page 4	Last two paragraphs of the introduction.
	**METHODS**				
4	**Study design and data sources**		Present key elements of the study design early in the article. Consider including a table listing sources of data for all phases of the study. For each data source contributing to the analysis, describe the following:		
		a)	Setting: Describe the study design and the underlying population, if possible. Describe the setting, locations, and relevant dates, including periods of recruitment, exposure, follow-up, and data collection, when available.	Pages 4, 5, and Table 1	Information about the GWAS studies is summarised in sections "data sources for inflammatory marker exposures" and "data source for female inflammatory condition outcomes". The studies from which the GWASs derive are also cited in table 1 for reference.
		b)	Participants: Give the eligibility criteria, and the sources and methods of selection of participants. Report the sample size, and whether any power or sample size calculations were carried out prior to the main analysis	Pages 4 and 5	In the methods section, we have described the eligibility criteria, data sources, and methods of selection of cohorts for the outcome.
		c)	Describe measurement, quality control and selection of genetic variants	Pages 4 and 5	As stated in the Methods section "data sources for inflammatory marker exposures", we prioritised GWASs with the largest sample size with many associated SNPs (at least 3). We did not perform independent sample size analysis, however, we concluded GWASs were sufficiently powered for the analysis given the large samples present.

		d)	For each exposure, outcome, and other relevant variables, describe methods of assessment and diagnostic criteria for diseases	Table 1	Further information is provided in the original cited GWAS publications included in this manuscript, as well as under section “selection of genetic variants as instrumental variables”.
		e)	Provide details of ethics committee approval and participant informed consent, if relevant	N/A	All original GWAS studies obtained ethics committee approval which is available in the relevant cited studies. Since we used de-identified, publicly available data, ethics approval was not required and was thus not relevant.
5	**Assumptions**		Explicitly state the three core IV assumptions for the main analysis (relevance, independence and exclusion restriction) as well assumptions for any additional or sensitivity analysis	Pages 5 and 6	This information is provided under section “selection of genetic variants as instrumental variables”. We outlined the three key assumptions (relevance, independence, and exclusion restrictions), as well as the methods for assessing them.
6	**Statistical methods: main analysis**		Describe statistical methods and statistics used		
		a)	Describe how quantitative variables were handled in the analyses (i.e., scale, units, model)	Pages 5 and 6	Described in sections “selection of genetic variants as instrumental variables” and “Two-sample MR analyses”.
		b)	Describe how genetic variants were handled in the analyses and, if applicable, how their weights were selected	Pages 5 and 6	Described in sections “selection of genetic variants as instrumental variables” and “Two-sample MR analyses”.
		c)	Describe the MR estimator (e.g. two-stage least squares, Wald ratio) and related statistics. Detail the included covariates and, in case of two-sample MR, whether the same covariate set was used for adjustment in the two samples	Page 6	Described in section “two-sample MR analyses”.
		d)	Explain how missing data were addressed	N/A	This is not applicable to the GWAS repository.
		e)	If applicable, indicate how multiple testing was addressed	Page 7	This information is provided under section “single SNP analyses” where Bonferroni method was applied to correct for multiple testing.
7	**Assessment of assumptions**		Describe any methods or prior knowledge used to assess the assumptions or justify their validity	Page 7	Our sensitivity analyses (single SNP analysis and leave-one-out analyses), tests for heterogeneity (Cochrane’s Q test and funnel plots), and horizontal pleiotropy are described in detail in the methods section under their relevant sections.

8	**Sensitivity analyses and additional analyses**	Describe any sensitivity analyses or additional analyses performed (e.g. comparison of effect estimates from different approaches, independent replication, bias analytic techniques, validation of instruments, simulations)	Page 7	This information is provided under sections: “single SNP analyses”, “leave-one-out sensitivity analyses”, “heterogeneity analyses” and “analysis of horizontal pleiotropy and outliers”.
9	**Software and pre-registration**			
	a)	Name statistical software and package(s), including version and settings used	Page 4	All statistical analysis was performed in RStudio v.2.0 using the “TwoSampleMR”, “MRPracticals”, and “MRInstruments” packages. This information is provided in the first paragraph of the methods section.
	b)	State whether the study protocol and details were pre-registered (as well as when and where)	N/A	The analysis plan is described in the methods section. The study protocol was not registered, as this was an analysis of publicly available data, and is thus not relevant.
	**RESULTS**			
10	**Descriptive data**			
	a)	Report the numbers of individuals at each stage of included studies and reasons for exclusion. Consider use of a flow diagram	Pages 4 and 5	This information is provided in sections “data source for inflammatory marker exposures” and “data source for female inflammatory condition outcomes”. There is more information provided on the relevant cited studies from which this data was extracted.
	b)	Report summary statistics for phenotypic exposure(s), outcome(s), and other relevant variables (e.g. means, SDs, proportions)	Pages 4, 5, and Table 1.	Summary statistics are available under sections “data source for female inflammatory marker exposures” and data source for female inflammatory condition outcomes”. This information is also provided in original publications from which the GWASs are extracted.
	c)	If the data sources include meta-analyses of previous studies, provide the assessments of heterogeneity across these studies	Page 9	Heterogeneity analyses (Cochrane’s Q and p-values) for significant exposures (MCP-1/CCL2 and IL-9) are summarised in Table 3. There was no statistically significant heterogeneity in any 2-SMR analyses (p>0.05), except for IL-2 and endometriosis which displayed statistically significant heterogeneity via the IVW method. These results are demonstrated in funnel plots for the associations between MCP-1/CCL2 and

					polycystic ovary syndrome, and IL-2, IL-9 and endometriosis (Figures 1D, 2D, and 3D).
		d)	For two-sample MR:<br>i. Provide justification of the similarity of the genetic variant-exposure associations between the exposure and outcome samples<br>ii. Provide information on the number of individuals who overlap between the exposure and outcome studies	Supplementary data file S3, 5	(i) We provide this information in Supplementary data file S3.<br>(ii) This information is provided in the section “data source for female inflammatory condition outcomes”.
11	**Main results**				
		a)	Report the associations between genetic variant and exposure, and between genetic variant and outcome, preferably on an interpretable scale	Supplementary data file 3	Associations between IVs and exposures as well as IVs and outcomes are presented in supplementary data file S3.
		b)	Report MR estimates of the relationship between exposure and outcome, and the measures of uncertainty from the MR analysis, on an interpretable scale, such as odds ratio or relative risk per SD difference	Pages 7, 8, and table 2	The associations of our results for primary univariable analyses are presented with causal estimates, odds ratios, and confidence intervals, in the first paragraphs of the results section, as well as in table 2.
		c)	If relevant, consider translating estimates of relative risk into absolute risk for a meaningful time period	N/A	This is not relevant to our study design.
		d)	Consider plots to visualize results (e.g. forest plot, scatterplot of associations between genetic variants and outcome versus between genetic variants and exposure)	Figures	These are all present in presented figures (also having subsections A, B, C, D) displaying forest plots, single SNP analyses, heterogeneity analyses, and funnel plots.
12	**Assessment of assumptions**				
		a)	Report the assessment of the validity of the assumptions	Pages 8 and 9	We assess the validity using sensitivity analyses and findings are presented under the sections “single SNP analyses”, “leave-one-out analyses”, “heterogeneity analyses”, and “horizontal pleiotropy and outilers”.
		b)	Report any additional statistics (e.g., assessments of heterogeneity across genetic variants, such as I^2, Q statistic or E-value)		
13	**Sensitivity analyses and additional analyses**				

		a)	Report any sensitivity analyses to assess the robustness of the main results to violations of the assumptions	Pages 8 and 9	We assess the validity using sensitivity analyses and findings are presented under the sections “single SNP analyses”, “leave-one-out analyses”, “heterogeneity analyses”, and “horizontal pleiotropy and outilers”.
		b)	Report results from other sensitivity analyses or additional analyses	N/A	No additional analyses other than what is presented herein.
		c)	Report any assessment of direction of causal relationship (e.g., bidirectional MR)	Figures	We assessed the direction of causal associations using scatter plots.
		d)	When relevant, report and compare with estimates from non-MR analyses	Pages 9-12	We compared these findings from observational studies throughout the discussion.
		e)	Consider additional plots to visualize results (e.g., leave-one-out analyses)	Figures	We included leave-one-out analyses to visualise results as forest plots.
	**DISCUSSION**				
14	**Key results**		Summarize key results with reference to study objectives	Page 9	This is provided in the first paragraph of the discussion section.
15	**Limitations**		Discuss limitations of the study, taking into account the validity of the IV assumptions, other sources of potential bias, and imprecision. Discuss both direction and magnitude of any potential bias and any efforts to address them	Pages 11 and 12.	This is comprehensively discussed in the second paragraph of the “Strengths and Limitations” section.
16	**Interpretation**				
		a)	Meaning: Give a cautious overall interpretation of results in the context of their limitations and in comparison with other studies	Page 13	This is discussed in the paragraph of the “conclusions” section.
		b)	Mechanism: Discuss underlying biological mechanisms that could drive a potential causal relationship between the investigated exposure and the outcome, and whether the gene-environment equivalence assumption is reasonable. Use causal language carefully, clarifying that IV estimates may provide causal effects only under certain assumptions	Pages 9 - 12	This is discussed throughout the discussion section.
		c)	Clinical relevance: Discuss whether the results have clinical or public policy relevance, and to what extent they inform effect sizes of possible interventions	Pages 9-13	This is discussed throughout the discussion and in the conclusions section
17	**Generalizability**		Discuss the generalizability of the study results (a) to other populations, (b) across other exposure periods/timings, and (c) across other levels of exposure	Page 12	This is discussed in the final paragraph of the “strengths and limitations” section.
	**OTHER INFORMATION**				

18	**Funding**	Describe sources of funding and the role of funders in the present study and, if applicable, sources of funding for the databases and original study or studies on which the present study is based	Page 1	This information is provided under the section “funding statement” on the title page.
19	**Data and data sharing**	Provide the data used to perform all analyses or report where and how the data can be accessed, and reference these sources in the article. Provide the statistical code needed to reproduce the results in the article, or report whether the code is publicly accessible and if so, where	N/A	This data is from a de-identified, summarised, and publicly available database known as the GWAS catalogue, which is noted throughout the manuscript.
20	**Conflicts of Interest**	All authors should declare all potential conflicts of interest	Page 1	This information is provided under the section “disclosure statement” on the title page. No authors disclosed any biases.

This checklist is copyrighted by the Equator Network under the Creative Commons Attribution 3.0 Unported (CC BY 3.0) license.

1. Skrivankova VW, Richmond RC, Woolf BAR, Yarmolinsky J, Davies NM, Swanson SA, et al. Strengthening the Reporting of Observational Studies in Epidemiology using Mendelian Randomization (STROBE-MR) Statement. JAMA. 2021;under review.

2. Skrivankova VW, Richmond RC, Woolf BAR, Davies NM, Swanson SA, VanderWeele TJ, et al. Strengthening the Reporting of Observational Studies in Epidemiology using Mendelian Randomisation (STROBE-MR): Explanation and Elaboration. BMJ. 2021;375:n2233.
